# Effects of decadal climate variability on spatiotemporal distribution of Indo-Pacific yellowfin tuna population

**DOI:** 10.1038/s41598-022-17882-w

**Published:** 2022-08-12

**Authors:** Yan-Lun Wu, Kuo-Wei Lan, Karen Evans, Yi-Jay Chang, Jui-Wen Chan

**Affiliations:** 1 Department of Environmental Biology and Fisheries Science, National Taiwan Ocean University, Keelung, Taiwan, R.O.C.; 2 Center of Excellence for Oceans, National Taiwan Ocean University, Keelung, Taiwan, R.O.C.; 3 CSIRO Oceans and Atmosphere, Castray Esplanade, Hobart, TAS 7001 Australia; 4 Institute of Oceanography, National Taiwan University, Taipei, Taiwan, R.O.C.; 5 National Applied Research Laboratories, Taiwan Ocean Research Institute, Taipei, Taiwan, R.O.C.

**Keywords:** Climate change, Ocean sciences, Phenology, Population dynamics

## Abstract

Spatial variations in tuna population and abundance are strongly linked to large-scale climate fluctuations, such as the Pacific decadal oscillation (PDO) and Atlantic multidecadal oscillation (AMO). However, the mechanisms underlying the association of climate indices with yellowfin tuna (YFT) abundance and habitat preference remain unclear. We analysed long-term longline fishery data for YFT and oceanic climate variability index data for 1971–2018. The standardized catch per unit effort (CPUE) of Indo-Pacific Ocean YFT was higher during negative AMO and positive PDO phases. In tropical Pacific Ocean, the trend of YFT habitat preference exhibited seesaw patterns because of the distinct environmental factors influenced by the PDO phase. The PDO changed the environmental parameters throughout the tropical Indian Ocean such that the habitat preference of YFT remained consistent throughout. However, the variations in habitat suitability did not correspond to the distribution or standardized CPUE of YFT throughout the Pacific Ocean during AMO events. Moreover, the changes in habitat suitability had a positive periodicity of 8–16 years with AMO in the Indian Ocean, but revealed opposite trends with the distribution or standardized CPUE of YFT. Our results provide sufficient information to distinguish the variations between PDO phase changing and YFT standardized CPUE/ habitat preference. Furthermore, the AMO phase shift period 60–100 years longer than that of the PDO (20–30 years), and models employing time series of fishery and environmental data must be extended the time period of our study to make the AMO match the fishery data more complete.

## Introduction

Climate change has caused shifts in species distributions in marine systems^1^. Spatiotemporal distribution models of top predators have been widely used to understand tuna population variation in environments changing as a result of climatic factors^2,3^. Tuna species are sensitive to the physical effects of climate variation on the marine environment, for example, changes to ocean temperature, and winds. Climate change has affected tuna fisheries by altering water temperatures^4^ and influencing marine productivity, marine organism distribution, and food web structures^5,6^. Changes in interannual and decadal climate patterns may explain the variations in the distribution and abundance of tuna. Interannual climate indices are mainly limited to use in analyses of adjacent basins, whereas multidecadal climate indices have wide-reaching teleconnections that affect large areas spanning multiple basins^7–11^.

Multidecadal climate indices have been more useful than interannual climate indices for understanding the processes underlying the bottom-up control of the pelagic ecosystem and the various life stages of top predators, including tuna species^7,8,10,12^. The Pacific decadal oscillation (PDO) index is the most prominent index of decadal variability in the North Pacific Ocean, and the PDO is considered a factor contributing to the surface warming hiatus that occurred in the late twentieth century because of fluctuations in the global mean temperature^13^. In the western and central Pacific Ocean, the PDO plays a bottom-up role in regulating bigeye tuna recruitment and abundance^7,12^. The Atlantic multidecadal oscillation (AMO) index, an index of linearly detrended North Atlantic Ocean temperatures, indicated a dominant influence of bluefin and yellowfin (*Thunnus albacores*; YFT) tuna abundance in ocean basins^7,8^. The North Pacific gyre oscillation (NPGO) is driven by region- and basin-scale variations in wind-driven upwelling and horizontal advection^14^; it has markedly influenced the top predator abundance and density-dependent competition in the Pacific Ocean^7,15^.

Yellowfin tuna (YFT) constitute a major target in the industrialized fisheries of the Indo-Pacific Ocean and are predominantly caught using purse seines and longline fishing^16,17^. In the 1960s, longline fisheries accounted for nearly 50% of fish caught globally^18^. Although purse seines have been the most prevalent type of gear used to target YFT since the 1970s, YFT can still be targeted through deep or shallow longline fishing^19^. These methods allow YFT to be caught in high quantities, making them the second most fished tuna species. Moreover, the endothermic nature of YFT allows them to migrate extensively as a top marine predator inhabiting tropical and subtropical pelagic water around the world’s three ocean basins^18^. YFT spend most of their time either within the surface mixed layer or at the top of the thermocline, where phytoplankton production is highly and epipelagic prey are concentrated^9,10,18^.

Several studies have demonstrated that multidecadal climate variabilities (i.e., AMO, PDO, and NPGO) play a pivotal role in global YFT population dynamics, including recruitment and distribution^7–15,20–22^. Numerous studies have also revealed strong links to climatic phenomena within a single basin, but few studies have focused on transoceanic phenomena. YFT catches in the Indo-Pacific Ocean account for over 50% of the total catches from longline fishing^7^. However, the influences of anthropogenic factors (e.g., fishery capture activity, over fishing) cannot be ignored because species populations change with the oceanic environment. Planque et al.^23^ reviewed how fishery exploitation can alter the structure of fish populations and thereby affect those populations’ ability to respond to climate variability and change. Fishery exploitation may alter the associations between climate indices and fish populations. However, marine scientists tend to suggest that either (1) “natural” climate variability or (2) the exploitation of fisheries is primarily responsible for fish population declines and the associated changes to ecosystem. However, in most cases, the effects of both climate and exploitation are probably substantial^23,24^.

According Wu et al.^7^, multidecadal climate indices influence the global YFT population. Decadal climate indices indicate a 0–5-year lag for the YFT population. However, whether the population exhibits variation during phase changes remains unclear. Therefore, in the present study, we investigated the influence of decadal climate patterns on the distribution and habitat preference of YFT in the Indo-Pacific Ocean by using long-term (1971–2018) longline fishery data. The objective of this study was to analyse the effects of the interactions between YFT and decadal climate variabilities and explore the process underlying the high phenotypic plasticity that mitigate the effects of climate change on top marine predators as YFT (Fig. S1).

## Result

### Large-scale climate indices and Indo-Pacific Ocean yellowfin tuna

**Figure 1:**
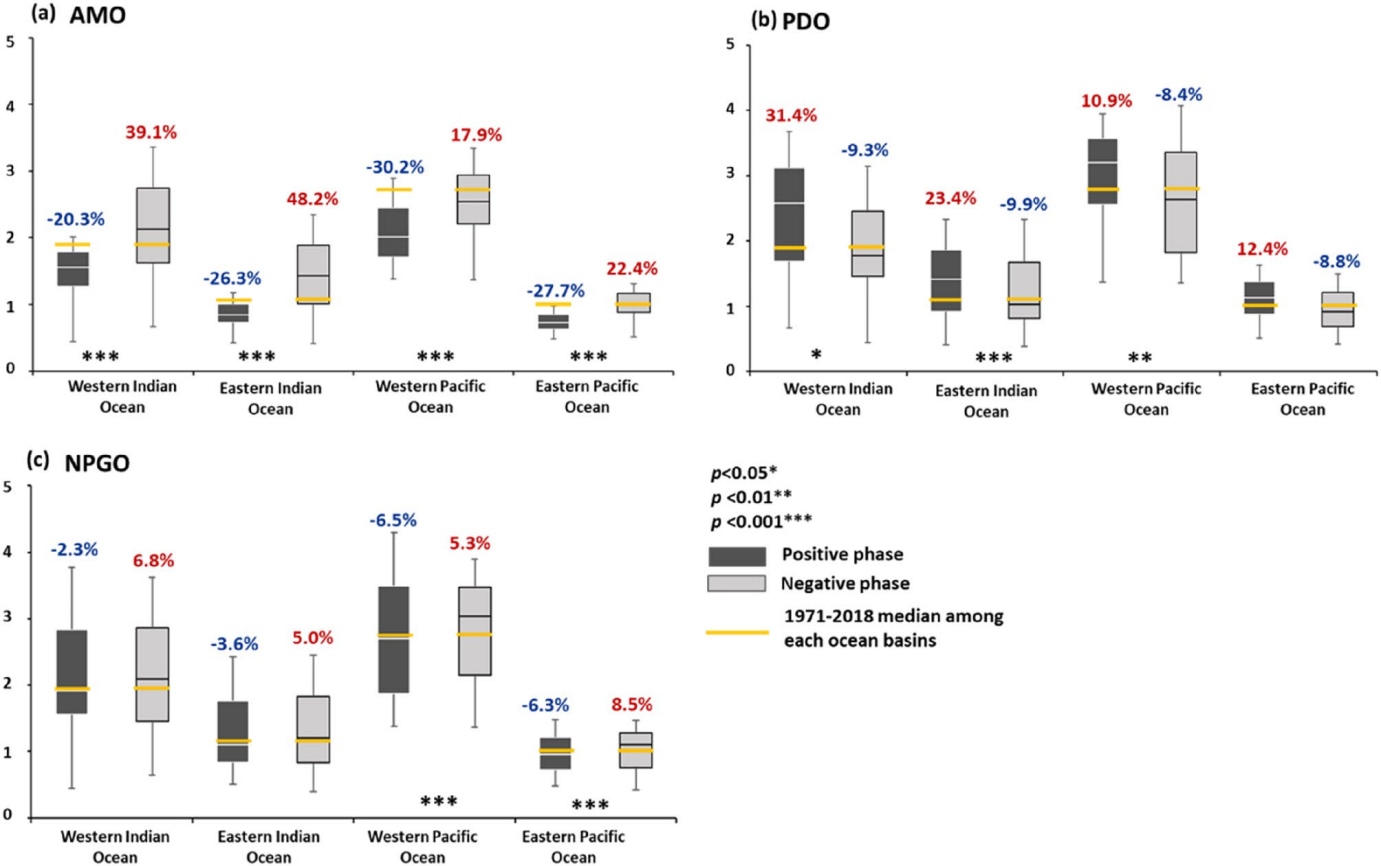
Box plot of each ocean basins’ CPUE values. (**a**) During AMO events. (**b**) During PDO events. (**c**) During positive and negative NPGO events. Values displayed in the figure represent the median increase (red) and decrease (blue) percentages as compared with the 1971–2018 median.

The standardized CPUE data for YFT from 1971 to 2018 were reorganized on the basis of different climatic events, including those of the AMO, PDO, and NPGO (Fig. 1). During the negative AMO phases, the median standardized CPUE for each basin was higher than it was during the positive phases, and all basins showed significant variation during the AMO phase change (*p* < 0.001; Fig. 1a). In the positive PDO phases, the median standardized CPUE was higher than in the negative phases (Fig. 1b); the highest CPUE values in the western Pacific Ocean and the Indian Ocean are 10.9, 31.4 and 23.4% higher respectively than their mean values. The aforementioned basins varied significantly during the PDO phase change; however, the eastern Pacific Ocean exhibited only slight differences. Only the Pacific Ocean exhibited any significant differences (*p* < 0.001) under positive or negative NPGO events (Fig. 1c).

**Figure 2:**
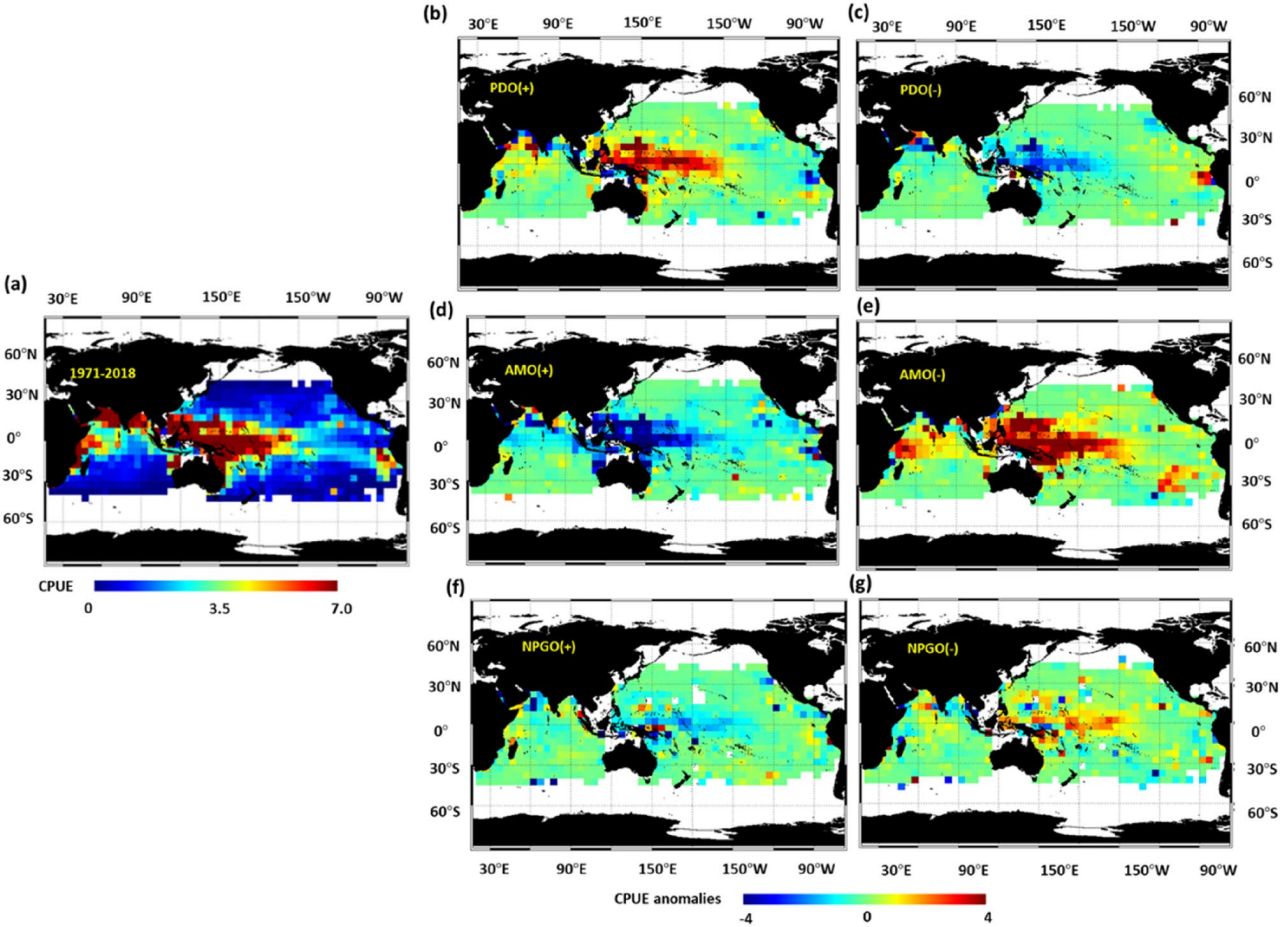
Yellowfin tuna spatial distributions. Distributions of yellowfin tuna CPUE anomalies from (**a**) 1971–2018 and positive (**b**) PDO, (**d**) AMO, and (**f**) NPGO phase years. Distributions from negative (**c**) PDO, (**e**) AMO, and (**g**) NPGO phase years. Figure created with Interactive Data Visualization Solution (IDL v 8.7) software. Software Resource: https://www.l3harrisgeospatial.com/Software-Technology/IDL.

The spatial distribution of CPUE data during 1971–2018 reveals that the CPUE was high (> 5.0 individuals/1000 hooks) in the western and central Pacific Ocean and Arabian Sea and off the eastern coast of Africa (Fig. 2a). The CPUE anomalies were positive during positive PDO events and negative AMO events (Fig. 2b, e). By contrast, the CPUE anomalies were negative during negative PDO events and positive AMO events (Fig. 2c, d). However, no clear trend in CPUE anomalies was observed during NPGO events (Fig. 2f, g). In accordance with the aforementioned information, we analysed the relationships between the habitat preference of YFT and the PDO and AMO.

### Environmental parameter changes during decadal climate index phase changes

To determine how marine environments vary during climate index phase changes, we plotted the spatial distributions of four environmental parameters (sea surface temperature [SST], sea surface height [SSH], sea surface salinity [SSS], mixed layer depth [MLD]) during climate event (Figs. S2–S5). During the positive PDO phases, SST was lower in the subtropical and western tropical Pacific Ocean and in the western Indian Ocean (Fig. S2a), whereas higher values extended from the eastern to the central Pacific Ocean. The SST data indicated opposite distribution patterns during the negative PDO phases (Fig. S2b). SSH and SSS changed dramatically in the central and western Pacific Oceans during the phase change period (Figs. S3–S4), but no obvious differences were detected in the MLD (Fig. S5). During positive AMO events, the SST and SSH were increased, and the MLD was greater throughout the entire Indo-Pacific Ocean; however, these variables exhibited the opposite patterns during the negative phases (Figs. S2, S3, and S5).

### Importance of environmental influence and habitat suitability of Indo-Pacific YFT

The four environmental parameters, SST, SSH, SSS, and MLD (in terms of continuous partial lease-squares regression [PLSR] results and variable influence on projection [VIP] scores), were also used to identify general environmental variations during the decadal climate events. The PLSR results suggest that SST is the most crucial environmental factor for YFT CPUE (VIP score = 0.72; Table 1), which may have closely followed the SST variation with climate conditions. SSH was the second most important environmental parameter (VIP score = 0.38) to the standardized CPUE of YFT. The third and the fourth most influential parameters were MLD (VIP score = 0.33) and SSS (VIP score = 0.27), respectively (Table 1). However, in the PLSR results, the VIP scores of categorical predictors were lower than 0.2; thus, the VIP score of each environmental parameter was used for weighting to construct the Indo-Pacific Ocean YFT habitat preference model.

**Table 1:** PLSR results for importance of environmental variables to YFT CPUE.

Variable importance in projection of standardized CPUE			
Variable	Category value	VIP	Importance
sst	–	0.718	1
ssh	–	0.378	2
mld	–	0.328	3
sss	–	0.267	4
year	1971–2018	0.15–0.009	5–19, 22, 25, 31–34, 36–64
month	1–12	0.134 ~ 0.002	10, 12, 20–21, 23–24, 26–30, 35

**Figure 3:**
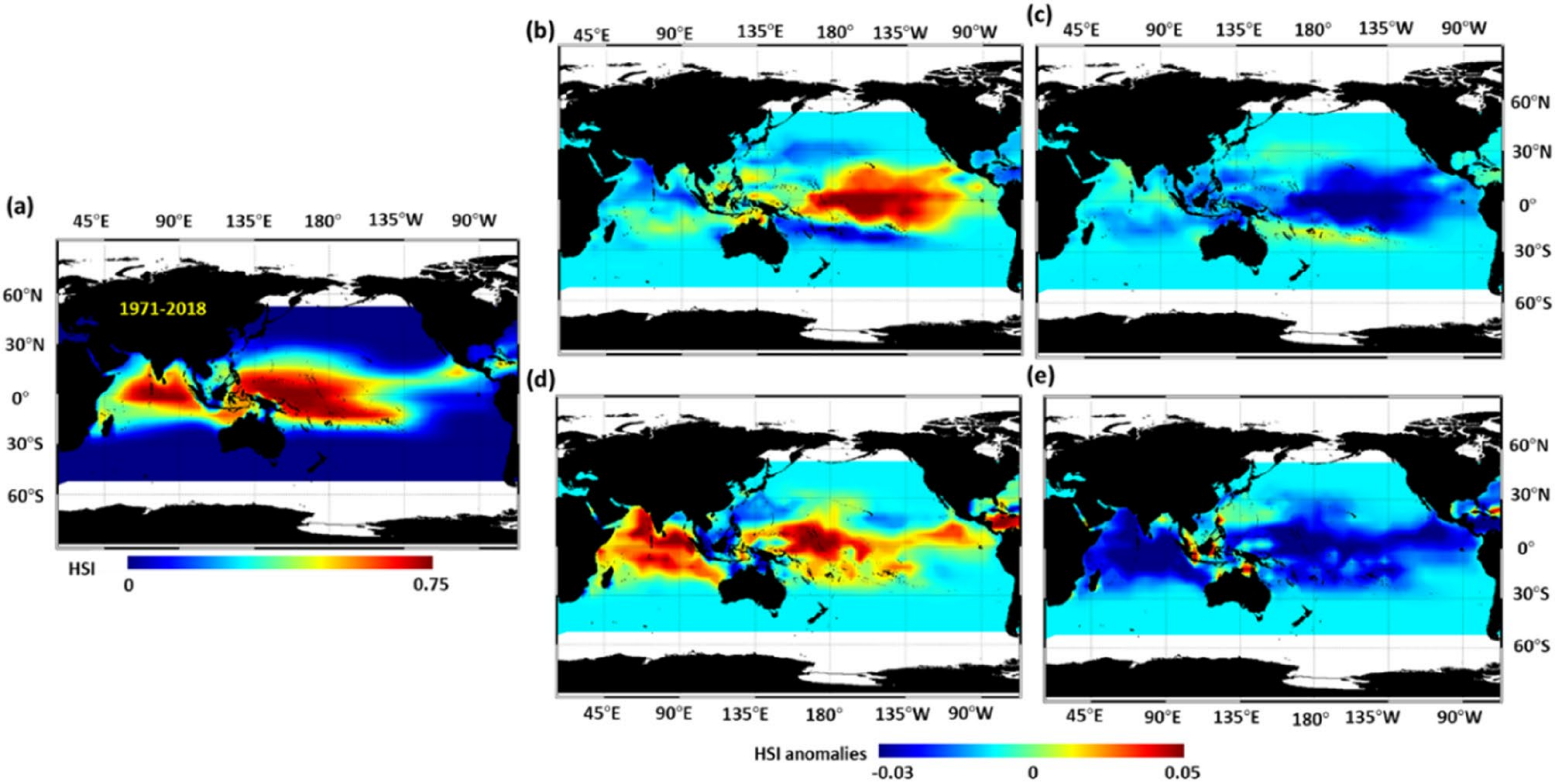
HSI spatial distributions anomalies. Distribution from (**a**) 1971–2018; (**b**) during positive PDO phase minus whole study period (1971–2018); (**c**) during negative PDO phase minus whole study period (1971–2018); (**d**) during positive AMO phase minus whole study period (1971–2018); (**e**) during negative AMO phase minus whole study period (1971–2018). Figure created with Interactive Data Visualization Solution (IDL v 8.7) software. Software Resource: https://www.l3harrisgeospatial.com/Software-Technology/IDL.

The suitable index (SI) analysis of the four environmental parameters indicated the preferred SST range of YFT to be 28.2–30.2 °C (SI > 0.75), with the highest proportion of 29.3 °C (Fig. S6a). The preferred SSH for Indo-Pacific YFT varied from 0.37 to 0.68 m (SI > 0.75), with the peak at 0.53 m (Fig. S6b). The preferred SSS range was 34.3–35.3 psu (SI > 0.75), with the medium at approximately to 35.1 psu (Fig. S6c). The preferred MLD values ranged from 22 to 45 m (SI > 0.75), peaking at 33.3 m (Fig. S6d). The weighted his in a geometric mean model (GMM) was high in the western and central Pacific and northern Indian Oceans over 1971–2018 period (Fig. 3a).

### Spatiotemporal variations of YFT habitat suitability during PDO and AMO phase changes

During the positive PDO phases, the YFT habitat suitability increased in the central tropical Pacific Ocean (5°N–12.5°S, 170°E–140°W) but decreased in the temperate part of the southern Pacific Ocean (12.5°S–27.5°S, 170°E–140°W) and the northern Indian Ocean (Figs. 3b and S7c). By contrast, the habitat suitability decreased throughout the Indo-Pacific Ocean during the negative PDO phases (Fig. 3c). The habitat suitability decreased throughout the Indo-Pacific Ocean during the negative AMO phase (Figs. 3e and S7e). Conversely, the habitat suitability increased during the positive AMO phases (Figs. 3d and S7d), especially in the northern Indian Ocean and tropical Pacific Ocean, where decreased CPUE was observed (Figs. 1a and 2d).

**Figure 4:**
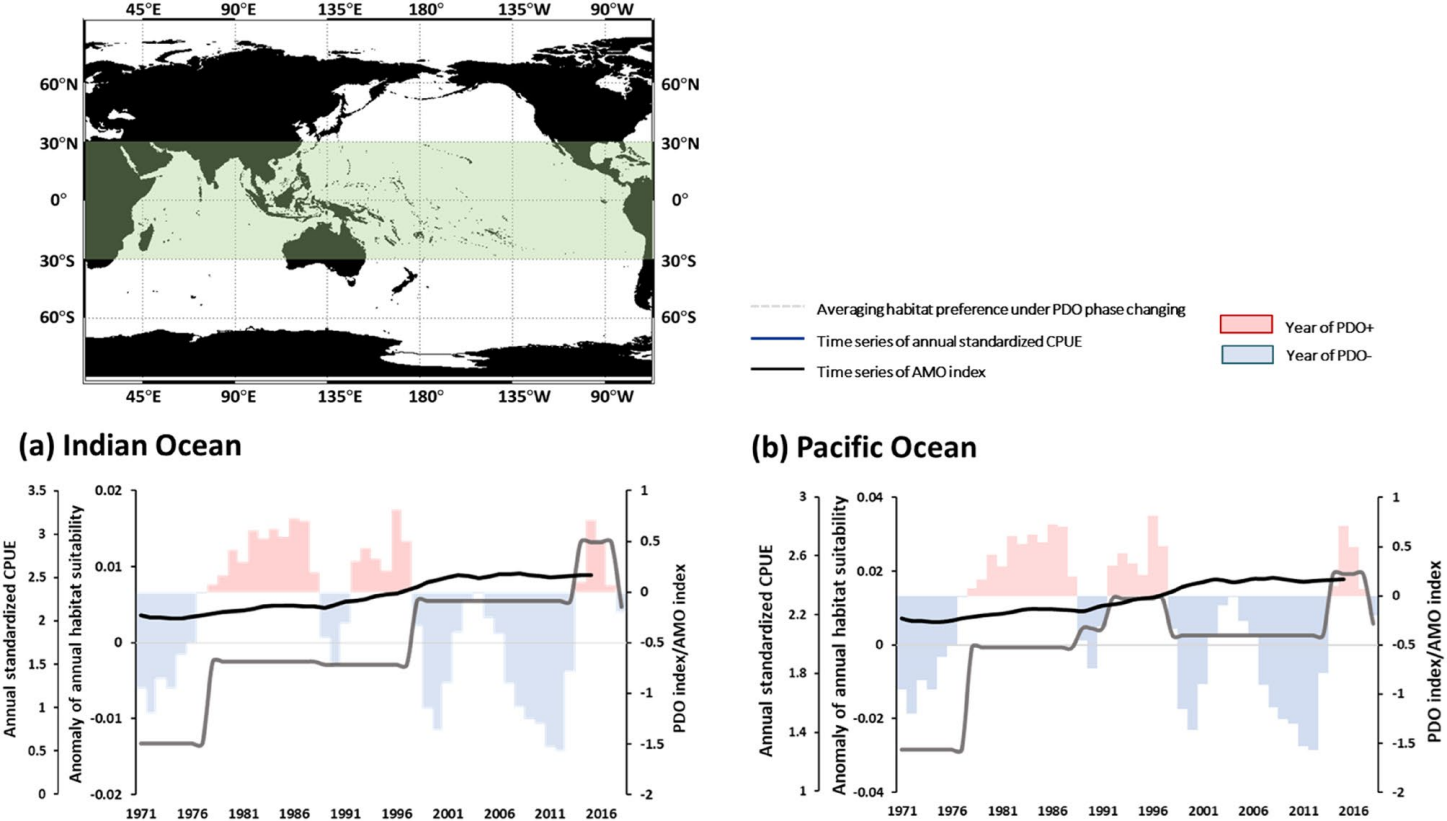
Average HSI anomalies of yellowfin tuna during AMO and PDO phase changes in designated areas.

**Figure 5:**
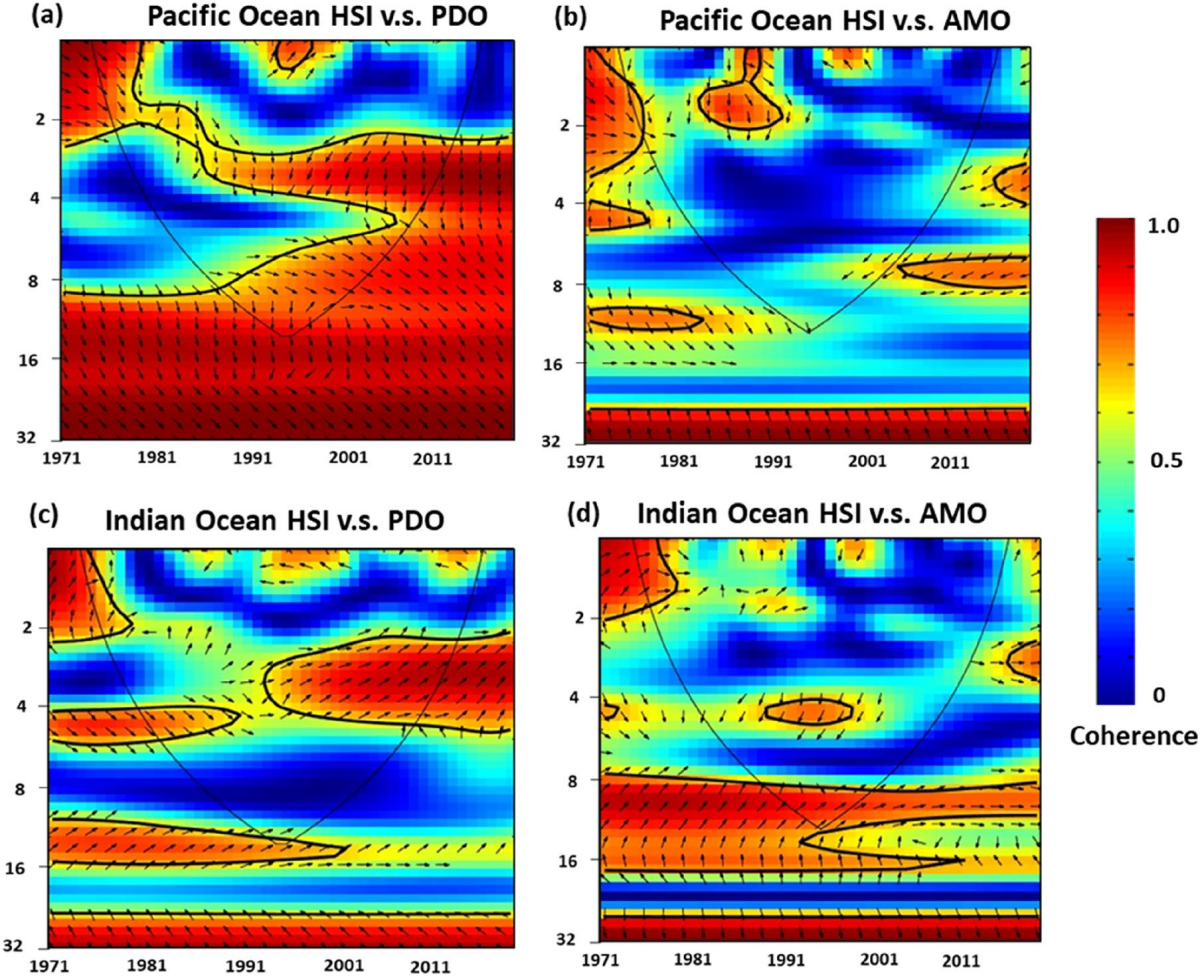
Cross-wavelet analyses of coherence between yellowfin tuna habitat suitability. (**a**) The HSI for yellowfin tuna in the Pacific Ocean compared with the PDO index. (**b**) The HSI for yellowfin tuna in the Pacific Ocean compared with AMO index. (**c**) The HSI for yellowfin tuna in the Indian Ocean compared with PDO index. (**d**) The HSI or yellowfin tuna in the Indian Ocean compare with AMO index. The solid black contours enclose regions of > 95% confidence, and the black lines indicate where the edge effects become salient. Red indicates high variability, and blue indicates low variability. Arrows indicate the phase relationships, with in-phase arrows pointing to the right and out-of-phase arrows pointing to the left.

Over 80% areas exhibited high variations of HSI spatial distribution in terms of tropical Indo-Pacific (30°N–30°S) YFT habitat preference modelling (Fig. 3). The temporal variations in habitat preference anomalies within the tropical Indian Ocean increased throughout the time period, and exhibit similar pattern with AMO (Fig. 4a). In the tropical Pacific Ocean, the annual values of habitat suitability exhibited an increasing trend from 1976 to 1996, 2011–2015 and decreased trends in 1995–2010 that evolved in a similar manner as the PDO index (Fig. 4b). Moreover, the cross-wavelet analysis between the HSI of the Pacific Ocean and PDO revealed positive or negative correlation during the study period (Fig. 5a). However, no clear associations between the AMO index and habitat suitability were observed in tropical Pacific Ocean during the study period (Fig. 5b). The interannual time series habitat suitability also revealed a positive correlation with the PDO index in the tropical Indian Ocean, with 4–6-year and 8–16-year periodicity (Fig. 5c). Furthermore, the wavelet analysis revealed that the habitat suitability was positively or negatively related to the AMO index, with 8–16-year periodicity during 1971–2018 in tropical Indian Ocean (Fig. 5d).

## Discussion

The present study provides valuable data demonstrating the effects of decadal climate variability on the spatiotemporal distribution of YFT. Numerous studies have also noted the influence of decadal climate variability on global tuna species abundance^7,8,22,26,27^. Decadal climate index phases changes can influence the marine environment and thus the standardized CPUE, spatial distribution, and habitat preference of YFT in the Indo-Pacific Ocean. Several studies have indicated that SST is the most crucial parameter influencing YFT abundance globally^4,28,29^. Arrizabalaga et al.^29^ showed SST explained 32.23% of the global variance in the habitat preference of commercial tuna species and suggested a preferred SST for YFT of up to 30 °C—the SI analysis in our study indicated a preferred SST of 28.2–30.2 °C. This result is consistent with the VIP score obtained through PLSR analysis (Table 2).

**Table 2:** Sources and specifications of global standardized CPUE, and climate index data.

		Item	Period			Resolution	Data source		Interpretation	
Standardized CPUE		Eastern Pacific Ocean	1971–2018			5°*5°	Inter American Tropical Tuna- Commission (IATTC) (https://www.iattc.org/)		The fleet which included fishing date, fishing ground, effort, and catches(number)	
		Western Pacific Ocean					Western and Central Pacific Fisheries Commission (WCPFC) (https://www.wcpfc.int/home)			
		Eastern Indian Ocean					Indian Ocean Tuna Commission (IOTC) (https://www.iotc.org/)			
		Western Indian Ocean								
Climate indices		AMO	1971–2018				https://www.esrl.noaa.gov/psd/data/correlation/amon.sm.data		Atlantic Ocean regional multi-decadal scale variation	
		PDO					https://www.ncdc.noaa.gov/teleconnections/pdo/		Broad-scale decadal scale variation in Pacific Ocean	
		NPGO					http://www.o3d.org/npgo/npgo.php		Broad-scale decadal scale variation in Pacific Ocean	

The results for SSH suggest its lower importance to HSI model accuracy that of SST^30^. Liu et al.^31^ reported that changes in thermocline depth were associated with SSH. An increase in SSH leads to an increase in MLD and therefore a deeper thermocline. Because YFT prefer to live in shallower thermocline or in layers shallower than the thermocline, the depth of the thermocline determines the depth that YFT schools inhabit, making SSH and MLD the second and third most critical factors, respectively, in YFT fisheries^30^. Although SSS did not achieve as high of a VIP score as the other environmental parameters, it is often used to model the global habitat preferences of commercially bred tuna species under the conditions of general ocean warming^4,29^. In summary, the environmental parameters investigated in this study affect the habitat preference of YFT by altering their daily vertical migration, feeding and spawning grounds, and recruitment locations^30,32–37^.

Previous studies have indicated that global YFT abundance is closely associated with decadal climate indices. Although studies have shown that interannual climate variabilities (e.g., El Niño–Southern Oscillation [ENSO]) exhibit strong correlation with spatial distributions and habitat preference of YFT in the Indo-Pacific Ocean^29,38^, some have focused on only the short-term fishing dynamics in a single ocean basin^7^. Another study reported that interannual climate indices (ENSO, Indian Ocean Dipole [IOD]) are mainly limited to use in analyses of adjacent basins^9^, whereas multidecadal climate indices have wide-reaching teleconnections that affect large areas spanning multiple basins. In this study, time series data indicated that the standardised CPUE and distribution were positively correlated with habitat suitability and influenced by the PDO in the Indo-Pacific Ocean. Specifically, the tropical Pacific Ocean was located at the edges of the warm and cool tongues^39^, and YFT habitat suitability exhibited a seesaw pattern affected by the long-term local fluctuations caused by PDO events. This result suggests that the PDO influences the standardized CPUE and distribution of YFT. Previous studies have also reported that the PDO causes global SST variation^40^ and influences the recruitment and abundance of YFT^7,22,41^. During the positive phases of the PDO, the Aleutian Low pressure system deepens and shifts southwards, and SSTs decrease in the central and western Pacific, cooling the winds and increasing the levels of nutrients and biological production more than negative PDO events^42^.

The PDO may be a major factor affecting the physical processes and subsequent responses of zooplankton community structures^43^. Olson et al.^44^ and Lan et al.^12^ have revealed major decadal dietary shifts among tuna species over a broad region of the Pacific Ocean, suggesting that the PDO affects the pelagic ecosystem by acting as a regulator of bottom-up control. The PDO significantly influences marine environments not only in the Pacific Ocean but also in the Indian Ocean by changing SSTs and the strength of the monsoon^45,46^. During positive PDO events, the subsurface (100–320 m) temperatures and thermocline depths throughout the Indian Ocean are lower and greater respectively^47^. Such changes may explain the findings that the CPUE and habitat suitability of YFT were increased in the Indian Ocean. Several studies have noted that tuna species abundance in the Indian Ocean is significantly affected by variations in SST, MLD, and net primary productivity, which are influenced by climate variability^10^.

The AMO index is a general measure of climate variability in the Atlantic Ocean on decadal and longer time scales. AMO-induced changes in Atlantic SSTs have regional effects on the SST in the northern hemisphere, Artic Sea ice, and fishery production in the northern Atlantic Ocean^48^. Through the teleconnection of the oceanic physical environment (e.g., strength of atmospheric vertical wind), the western tropical Pacific Ocean and Indian Ocean SST have been noted to be significantly influenced by the AMO^49,50^. Wu et al.^7^ reported that the AMO influenced the abundance of YFT in not only the Atlantic Ocean but also the global ocean, with a periodicity of 8–16 years. In addition, the fishing vessel dynamics and habitat suitability in the Atlantic Ocean are affected by AMO events^8,41^. In the present study, the standardized YFT CPUE was observed to be higher during negative AMO phases Indo-Pacific Ocean; however, the changes in habitat suitability under AMO phase changing did not correspond to the distribution or standardized CPUE of YFT Pacific Ocean (Fig. 4b). Although the changes in habitat suitability had a positive periodicity of 8–16 years with AMO in the Indian Ocean (Fig. 5d), but revealed opposite trends with the distribution or standardized CPUE of YFT (Figs. 1a, 2d, e). It could not clarified the influence of the AMO on the marine environment or ecosystem through our present analysis. The AMO phase shift period 60–100 years longer than that of the PDO (20–30 years), and models employing time series of fishery and environmental data must be extended to consider diverse gear types and fishing strategies to simulate animal responses to spatially heterogeneous biotic and abiotic conditions during AMO phases^8^.

Furthermore, studies have suggested that great care must be taken to distinguish the low-frequency changes associated with natural oceanic oscillations from anthropogenic changes^25^. We suggest two major reasons to distinguish the significant negative correlation between the AMO index and long-term longline yellowfin tuna fishery data. First, the increase in longline fishing efforts caused the overexploitation observed throughout the Indo-Pacific Ocean^51^. For example, the number of yellowfin tuna caught using purse seines (targeting immature yellowfin tuna) has increased since the 1980s. The increased use of purse seines may have reduced immature tuna abundance and even caused recruitment decline^22,52^. The continual decreases in longline fishery tuna species catches since 1980s coincide with the AMO phase shift that occurred in the 1990s and 2000s, resulting in the significant and strong correlations discovered in the time series analysis. Second, ocean warming has already affected global fisheries, including the populations of tropical and temperate tuna species^4,28^. The increasing SST levels caused by ocean warming since the 1980s coincide with the AMO phase shift that occurred in the 1990s and 2000s, yielding high correlation with tuna abundance in the time series analysis.

## Conclusion and remarks

**Figure 6:**
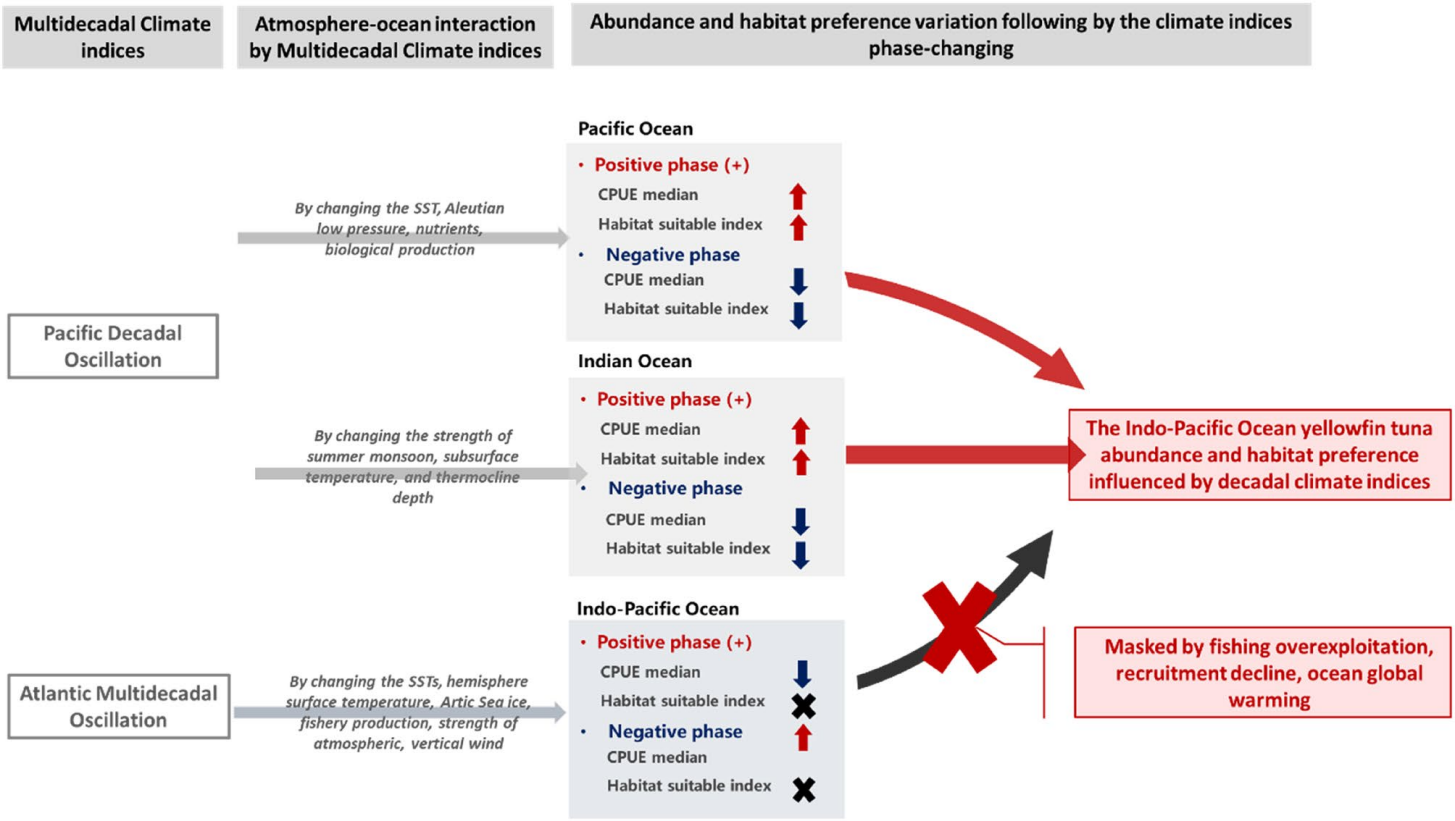
Mechanism by which multidecadal climate variability affects Indo-Pacific Ocean yellowfin tuna abundance and habitat preference.

Multidecadal climate variabilities affect the distributions of tuna species, and the standardized CPUE and habitat preferences of YFT are significantly influenced by PDO phase changes in the Indo-Pacific Ocean. The PDO changed the environmental parameters of the whole Indo-Pacific Ocean such that the habitat preference of yellowfin tuna was consistent throughout. In the present study, the standardized YFT CPUE was observed to be higher during negative AMO phases Indo-Pacific Ocean; however, the changes in habitat suitability did not correspond to the distribution or standardized CPUE of YFT (Fig. 6). Although heterogeneous biotic and abiotic conditions would affect under our changing PDO phase hypothesis, PDO had several times phase changing during our study periods. This provides us sufficient information to distinguish the variations between PDO phase changing and YFT standardized CPUE/ habitat preference. In conclude, the time period of our study might be the mostly barriers to realize the mechanism between AMO, biotic and abiotic conditions, and YFT abundance.

To be worth the mentioned that over-exploitation, variation of recruitment and ocean warming would both effect the YFT under our PDO and AMO phases changing hypothesis. Although predictions of the outcomes of the climate change–induced habitat shift of tuna species have becomes more positive, studies on the abundance and distribution of top predators at each life stage in marine ecosystems remain rare^7,12,21,22^. More complex mechanisms explaining the adaptive capacity, recruitment, and migration of top predators during their various life stages. We intend to include data on the diverse gear types used in tuna fishery to capture differences between tuna life stages in our subsequent research. Furthermore, comprehensive research informed by data on fisheries for species at other trophic levels will be considered to evaluate how climate change affects the top-down control of yellowfin tuna.

## Materials and methods

### Framework of dataset

We first analysed the spatial distribution and median standardized catch per unit effort (CPUE) of the long-term fisheries and climate data (Fig. S1a–c) to see how YFT abundance changes during distinct climatic phases. We also established the YFT HSI according to changes in the climate variability phases (Fig. S1d–g). Through habitat suitability analysis, we identified the preferred habitats and crucial environmental parameters of YFT during distinct climate events. Moreover, we applied wavelet analysis to further explore the mechanism underlying the changes in the YFT standardized CPUE and habitat suitability using the decadal climate indices (Fig. S1h, i).

### Data

#### Longline fishery catch data

Publicly available longline fishery data for the Atlantic, Indian, and Pacific Oceans for 1971–2018 were obtained from three tuna regional fishery management organizations (tRFMOs; Table 2 included the linking of data resource). Fishery data generated or analysed during this study are included in this published article (details see the supplementary file for dataset). YFT is divided into four stocks, each of which is currently managed by a separate tRFMOS. The effort and catch in the eastern and western parts of both the Indian Ocean and Pacific Ocean differed. The western Indian Ocean and western Pacific Ocean accounted for over 50% of the effort and catch in each ocean^18,41^. On the basis of this information, we classified the Indo-Pacific Ocean into four regions. YFT catch (species by number, depending on the fleet) and operations (number of hooks and area coordinates) data from each tRFMO were compiled with a 5° spatial resolution. The monthly nominal CPUE was calculated as the number of individual fish captured per 1000 hooks in four regions (i.e., the western and eastern Indian and Pacific Oceans) as they related to the currently accepted stocks of the regions, as identified by the tRFMOs (Table 2; supplementary file for dataset). Furthermore, standardization of both CPUE variables was required because the nominal CPUE can vary substantially in space and time depending on gear efficiency, gear configuration, and targeting practices (principally driven by market trends such as fishing primarily YFT or bigeye tuna) among other factors. We adopted the standardized CPUE as the YFT abundance indicator to investigate YFT variation over time under climate change.

The nominal CPUE was categorized according to each grid (latitude × longitude; Fig. S1c), and the YFT spatial distribution was plotted to evaluate how YFT migration changed during climate events. The main YFT effort and YFT catch data were derived from Japan, Taiwan, Korea, and China in Indian Ocean. In eastern Pacific Ocean, the effort and catch were mainly belonged from Japan. It took the occupied over 60% YFT effort and 73% YFT catch in IATTC. There are many countries capture the YFT in western and central Pacific Ocean, the effort from Taiwanese and Japanese flag, take 59 percent. The other countries included China and Korea, etc. Moreover, above-mentioned countries take 90 percent of YFT catch in WCPFC.

#### Climate indices

The indices of multidecadal climate phenomena from the same period (1971–2018), that is, the AMO, PDO and NPGO, were compiled and calculated on a monthly time-scale, where the original data were smoothed (Table 2).

1. The AMO is a natural multidecadal variability in oceanic and atmospheric temperatures, with a range of 0.4 $^\circ$C and a periodicity of 60–100 years. However, our fishery data spanned only 57 years. Notably, an AMO phase changed occurred during 1997 and 1998. In this study, we retrieved AMO index data that had been extended and reconstructed from SST starting in 1948 and averaged the area over the North Atlantic (0°N–70°N)^53^.
2. The PDO index explains crucial climate fluctuations over the North Pacific. This index is defined by the leading principal component of SST anomalies north of 20°N. A noteworthy feature of the PDO index is the PDO’s extended periods (2–3 decades in duration) of predominantly positive or negative deviation from the long‐term mean. Little is known about the mechanisms underlying these periods^39^.
3. The NPGO is the second most dominant mode of variability in the SSH and SST anomalies of the northeastern Pacific Ocean (25°–62°N, 180°–250°E)^15^. The NPGO index reflects changes in the North Pacific gyre circulation and key physical and biological ocean variables, including SST, SSS, SSH, abundance of nutrients, and chlorophyll-a^15^.

#### Environmental variables

Monthly environmental data for the period of 1971–2018 were retrieved from the Asia–Pacific Data Research Center. These environmental data were analyzed to determine how the local marine environment changes during the phase changes of the decadal climate variabilities. Key environmental variables of potential relevance to YFT, including SST, SSH, MLD, and SSS, were obtained from the European Centre for Medium-Range Weather Forecasts (ECMWF) Ocean Reanalysis System 5 (ORAS5) with 1° spatial resolution^54^. We built up our environmental data resource by obtaining the ECMWF system from Asia–Pacific Data-Research Centre (APDRC). The ECMWF ORAS5 system is a new global eddy-permitting ocean and sea-ice packages for the reanalysis and modelling of oceanic variability in SST and SSH. The system generates accurate results, as verified against independent observational data sets. The data associated with each variable were spatially aggregated to a 5° resolution to align with the resolution of the catch data.

### Data analysis

#### Standardization of nominal CPUE

Standardization of CPUE was required because nominal CPUE can vary spatiotemporally with targeting practices and gear configuration, which can influence fishing efficiency^55^. For example, when standardizing the effort of longline gear targeting tuna, we must consider that the depth of the gear has increased over time as fishermen began targeting bigeye tuna, which are generally found at greater depths in the water column. The standardized CPUE was visualised for each ocean basin on Q–Q plots (Fig. S1b) and used to generate a YFT abundance box plot and to perform PLSR and habitat suitability analyses for the conditions associated with climate index phase changes. A generalized linear model was used to standardize CPUE, with the main effects considered being year, month, longitude, latitude, and the catch rates of albacore and bigeye tuna, in accordance with the following equation:

1 $$\begin{aligned} {\text{Log }}\left( {CPUE + c} \right) & = \mu + {\text{ year }} + {\text{ month }} + {\text{ latitude }} + {\text{ longitude}} \\ & \quad + {\text{ albacore catch rate }} + {\text{ bigeye catch rate }} + \varepsilon \\ \end{aligned}$$

where *CPUE* is the nominal CPUE of yellowfin tuna, μ is the intercept, and ε is a normally distributed variable with a mean of 0. Because the log-link function cannot handle zero values, a small value (10% of the overall mean nominal catch rate) was added to *CPUE* in accordance with t standardization procedures previously used for longline species^56,57^.

#### Nominal yellowfin tuna CPUE anomalies

We calculated the nominal YFT CPUE for each grid (latitude × longitude; Fig. S1c). Specifically, we identified the anomalies for each climatic event to determine how spatial distribution varies during such climatic events. The spatial distribution of nominal YFT CPUE anomalies during different climatic events in the Indo-Pacific Ocean were calculated as follows:

2 $$CPUEA_{ij} = \frac{{\mathop \sum \nolimits_{1}^{n} CPUE_{ij} }}{n} - \frac{{\mathop \sum \nolimits_{1971}^{2018} CPUE_{ij} }}{48}$$

where *n* denote the number during the climate indices phases. The number during each climate event was recorded and is reported in Table S1. The terms *i* and *j* represent longitude (60°–290°) and latitude (42.5°N–42.5°S), respectively.

#### Pearson correlation

We separated the standardized CPUE time series data into negative and positive cases depending on the phase of decadal climate indices. We used the Pearson correlation to analyse the relationship between the standardized CPUE during positive and negative phases. The sample size (number of years) for each case (positive or negative phase of each variability) are listed in Table S1. Although one study demonstrated that sample size of 25 years or more is sufficient for the Pearson correlation because of limited study periods, the sample size of some cases was only 18 years^58^. Weaver and Koopman (2014), however, observed that a normal approximation is more accurate for samples sizes of more than 10 years than the Pearson correlation is^59^. Yet, this approach does not address the problem of computing valid p-values from correlation analyses when the assumption of independence is violated. To address this, we performed data analysis using the following steps. The Pearson correlations in Statistica 8.0 to determine whether the standardized CPUE of YFT changed significantly (i.e., *p* < 0.05) during the climate index phase changes (Fig. S1b).

#### PLSR

Because each environmental parameter has a different degree of influence on YFT, we applied PLSR, which is a technique that reduces the predictors to a smaller set especially when the predictors are highly collinear. PLSR was used to investigate the influence degree of the environmental parameters on the standardized CPUE (Fig. S1f)^60^. We also adopted the top 20% of the YFT standardized CPUE instead of the whole YFT data set to examine the preferred environmental characteristics of YFT. PLSR is a multivariate linear regression method to develop models of the correlations of categorical (here, year and month) and continuous (the four environmental parameters) predicators with response variables (the top 20% yellowfin tuna standardized CPUE) for a given set of samples. PLSR could distinguish which of the categorical and continuous predicators were most critical for the response variable. The sorting of each predictor, especially the continuous predicators (environmental parameters), allowed us to construct a complete habitat preference model in the next stage. PLSR thus provides information about the variables’ correlation structures as well as their structural similarities or differences.

#### VIP

VIP scores were determined to represent the influence of individual categorical (year, month) and continuous (the four environmental parameters) predictors on the PLSR model of YFT CPUE in the Indo-Pacific Ocean (Fig. S1f). The VIP scores were calculated as the weighted sum of squares of the PLSR weights, which involved considering the explanatory power of each latent variable. In the weighted sum of square is defined as where the response variable is the top 20% of YFT standardized CPUE and W (VIP scores) is the weight variable. The VIP score (weight variable) provide a useful measure for identifying which variables explained the greatest amounts of variance in the outcome (top 20% of standardized CPUE). The weighting (VIP score) of each environmental parameters was then applied for the habitat suitability analysis. The PLSR analyses were conducted using Statistica 8.0.

### SI calculation for the four environmental parameters

The purpose of SI calculation was to quantify the environmental preferences of YFT under the conditions of climate index phase changes (Fig. S1e). On the basis of the monthly frequency distribution of standardized CPUE, an SI for each environmental variable was calculated as follows:

3 $$SI_{ymij} = \frac{{CPUE_{ymij} }}{{CPUE_{max} }}$$

where ${CPUE}_{ymij}$ is the relative abundance index at longitude *i* and latitude *j* (the center of each 5 × 5 grid) in month *m* and year *y*. ${CPUE}_{max}$ is the maximum standardized CPUE for each month. The SIs of the environmental variables calculated using Eq. (3) were used as observed values to fit SI models with the midpoint of each environmental variable’s class interval. Each value was divided by the maximum frequency value to obtain a relative frequency distribution before being calculated using the following formulas:

4 $${\text{SI}}_{{\text{i}}} = {\text{Exp}}\left[ {{\upalpha }\left( {X_{i} - {\upbeta }} \right)^{2} } \right]$$

$\mathrm{\alpha }$ and $\upbeta$ are calculated through the least-squares method of minimizing the residuals between the predicted and observed SIs, and *i* denotes the environmental variables considered^61^. Using the SI values from each environmental parameter, we could obtain detailed information on Indo-Pacific Ocean YFT habitat preference.

### Habitat suitability models

HSI models are used to estimate habitat suitability for given species on the basis of one or more relevant habitat variables (e.g., four environmental parameters used in the study)^62,63^. Here, the HSI is the univariate model output with a value between 0 and 1. The associations between standardized CPUE and each of the environmental variables was converted into a curve of SI, which was continuous and ranged between 0 and 1. The most common empirical models were used to develop the optimal HSI model, including the arithmetic mean model (AMM)^61,63,64^ and GMM^63,64^. The GMM has an advantage over the AMM in that it is less affected by extreme values in skewed distribution. Therefore, we employed a GMM to develop our optimal HSI model^63,64^. We used the weighting results for habitat suitability (the VIP scores) for each environmental parameter into our HSI models as follows:

5 $${\text{HSI}}_{{{\text{GMM}}}} = \left( {\mathop \prod \limits_{n - 1}^{n} SI_{i} *W_{i} } \right)^{{1/\sum W_{i} }}$$

### Yellowfin tuna HSI anomalies

We calculated the HSI of YFT for each grid (latitude × longitude). Specifically, we identified the anomalies during each climatic event to determine how the spatial distribution of HSI varies during such events (Fig. S1g) by using the following equation:

6 $$HSIA_{ij} = \frac{{\mathop \sum \nolimits_{1}^{n} HSI_{ij} }}{n} - \frac{{\mathop \sum \nolimits_{1971}^{2018} HSI_{ij} }}{48}$$

### Cross-wavelet coherence analyses

We used wavelet analysis to investigate how environmental variations caused by the decadal climatic events affect the habitat suitability and standardized CPUE of YFT (Fig. S1i). Fourier spectral analysis is commonly used to analyse periodicity in time series data but assumes that the time series is stationary. The time series of climate indices and fishery data are not stationary. We used wavelet analysis because it requires no such assumption (7). The wavelet transformation is based on a convolution of a time series ${y}_{n}$ (*n* = 0…, *N* − 1, with equal spacing $\delta t$) and a wavelet function. The Morlet wavelet is the most popular complex wavelet used in practice and is defined as follows:

7 $$\psi_{0} (\eta ) = \pi^{ - 1/4} e^{{i\omega_{{0}} \eta }} e^{{ - \frac{1}{2}\eta^{2} }}$$

where $\eta$ is a dimensionless time parameter and $\omega_{0}$ is a dimensionless frequency used to balance time and frequency localisation. The wavelet transform of ${y}_{n}$ is calculated as follows:

8 $${\text{W}}_{{\text{ n}}}^{{\text{y}}} {(}s) = \sqrt {\frac{{{\delta t}}}{s}} \sum\limits_{{n^{\prime} = 1}}^{N} {y_{{n^{\prime}}} } \psi_{0} \left[ {(n^{\prime} - n)\frac{{{\delta t}}}{s}} \right]$$

where *s* is a scale such that $\eta$ = *st*. By varying *s*, the wavelet can by extended through time. A 5% significance level was set and based on 1000 bootstrap simulations with a spectral synthetic test^65^. The autoregression coefficient was empirically obtained from the time series data. Subsequently, cross-wavelet coherence and phase analyses were used to investigate the relationships between PDO or AMO events and the HSI of yellowfin tuna in the Indo-Pacific Ocean.

Cross-wavelet coherence and phase analyses represent cross-correlations normalised to the power of a single process and are thus not biased by the power of any single series^66^. We defined the cross-wavelet transformation of the two series $x_{n}$ and $y_{n}$ to be ${\text{W}}_{ \, }^{XY} = {\text{W}}_{ \, }^{X} {\text{W}}_{ \, }^{Y*}$, where * denotes a complex conjugation. The wavelet coherence was defined as follows:

9 $$R_{n}^{2} (s) = \frac{{\left| {S(s^{ - 1} {\text{W}}_{{\text{ n}}}^{XY} (s))} \right|^{2} }}{{S(s^{ - 1} ({\text{W}}_{{\text{ n}}}^{X} (s))^{2} \cdot S(s^{ - 1} {\text{W}}_{{\text{ n}}}^{Y} (s))^{2} }}$$

where *S* is a smoothing operator based on a running average.

The wavelet coherence phase was calculated as follows:

10 $$\varphi_{n} (s) = \tan^{ - 1} \left( {\frac{{{\text{Im}} aginary\left\{ {S\left( {s^{ - 1} {\text{W}}_{{\text{ n}}}^{XY} \left( s \right)} \right)} \right\}}}{{{\text{Re}} al\left\{ {S(s^{ - 1} {\text{W}}_{{\text{ n}}}^{XY} (s))} \right\}}}} \right)$$

where both $R_{n}^{2} (s)$ and $\phi_{n} (s)$ are functions of the time index *n* and scale *s*. Several studies have detailed the mathematics underlying such analyses^66,67^. The wavelet transform has edge artifacts because the wavelet is not completely localised in time, and the finite nature of such images gives rise to edge artifacts in reconstructed data. Therefore, a cone of influence can be introduced in which edge effects cannot be ignored^66^.

## Supplementary Information

Supplementary Information 1.

Supplementary Information 2.
