# Supplementary material for: Effects of decadal climate variability on spatiotemporal distribution of Indo-Pacific yellowfin tuna population
Source: Sci Rep. 2022 Aug 12;12:13715. doi: 10.1038/s41598-022-17882-w (PMC9374684; doi:10.1038/s41598-022-17882-w)
Supplement: Supplementary file 1 — Supplementary Information 1. [file 41598_2022_17882_MOESM1_ESM.docx]

**Effects of decadal climate variability on spatiotemporal distribution of Indo-Pacific yellowfin tuna population**

Yan-Lun Wu *^a^*, Kuo-Wei Lan* ^a,^*^b^* , Karen Evans *^c^*, Yi-Jay, Chang *^d^*, Jui-Wen Chan *^e^*

*^a^ Department of Environmental Biology and Fisheries Science, National Taiwan Ocean University, Keelung, Taiwan, R.O.C.*

*^b^ Center of Excellence for Oceans, National Taiwan Ocean University, Keelung, Taiwan, R.O.C.*

*^c^ CSIRO Oceans and Atmosphere, Castray Esplanade, Hobart, TAS, 7001, Australia*

*^d^ Institute of Oceanography, National Taiwan University, Taipei, Taiwan, R.O.C*

*^e^ National Applied Research Laboratories, Taiwan Ocean Research Institute, Taipei, Taiwan, R.O.C.*

Corresponding author: Kuo-Wei Lan

Email: k[wlan@mail.ntou.edu.tw](mailto:wlan@mail.ntou.edu.tw)

Tel: 886-2-24622192 ext. 5027

Table S1. Year affected by decadal climate phase changes.

|  | **Positive phase year** | **Negative phase year** |
| --- | --- | --- |
| **Atlantic Multidecadal Oscillation (AMO)** | 1998-2015  (Total: 18 years) | 1971-1997  (Total:27 years) |
| **Pacific Decadal Oscillation (PDO)** | 1977-1981, 1983-1987, 1992-1993, 1995-1997, 2003, 2014-2016  (Total: 19 years) | 1971-1976, 1982, 1988-1991, 1994, 1998-2002, 2004-2013, 2017-2018  (Total: 29 years) |
| **North Pacific Gyre Oscillation (NPGO)** | 1971, 1973, 1975-1978, 1984, 1987-1989, 1998-2004, 2007-2013  (Total: 24 years) | 1972, 1974, 1979-1983, 1985-1986, 1990-1997, 2005-2006, 2014-2018  (Total: 24 years) |


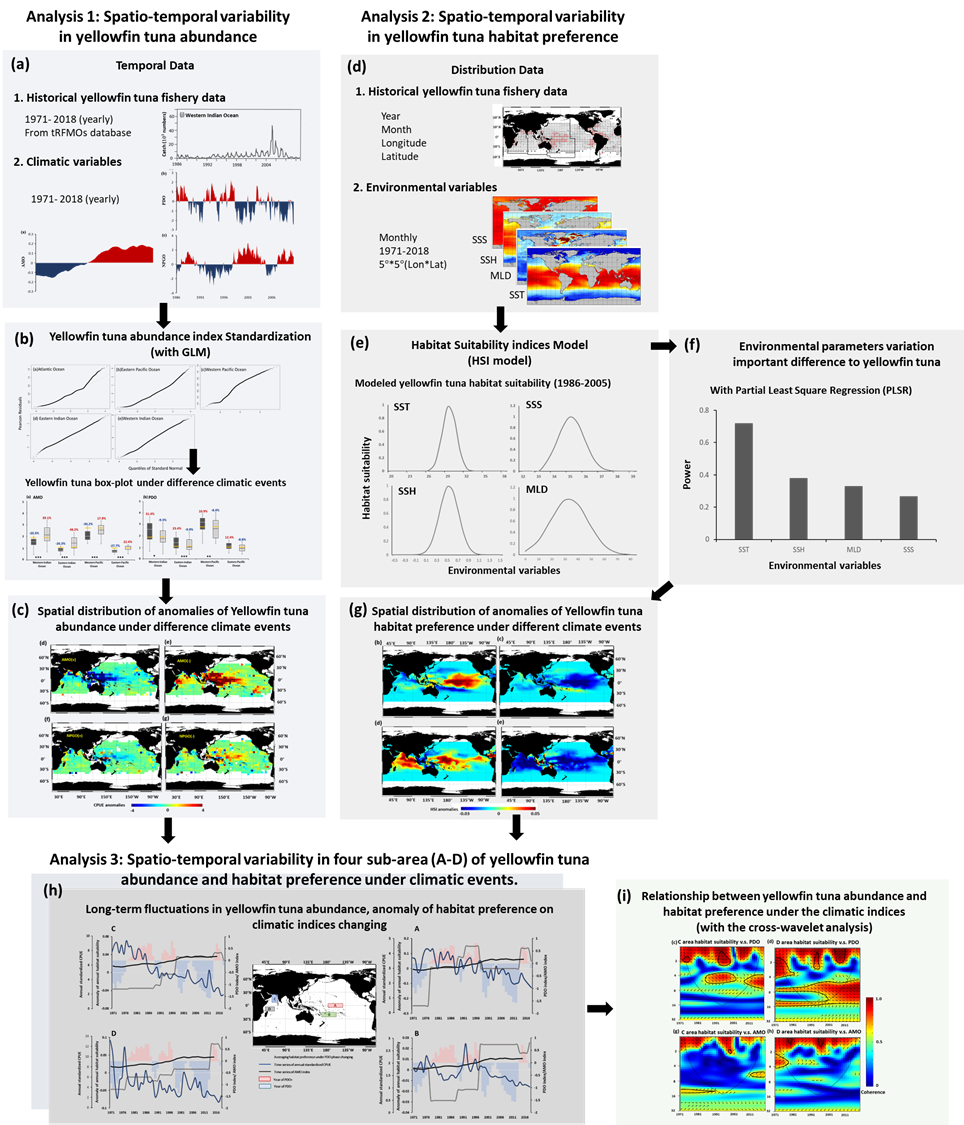


Fig. S1 Summary of the three-steps analysis conducted in this study. SST: sea surface temperature, SSH: sea surface height, SSS: sea surface salinity, MLD: mixed layer depth. Figure created with Interactive Data Visualization Solution (IDL v 8.7) software. Software Resource：<https://www.l3harrisgeospatial.com/Software-Technology/IDL>.


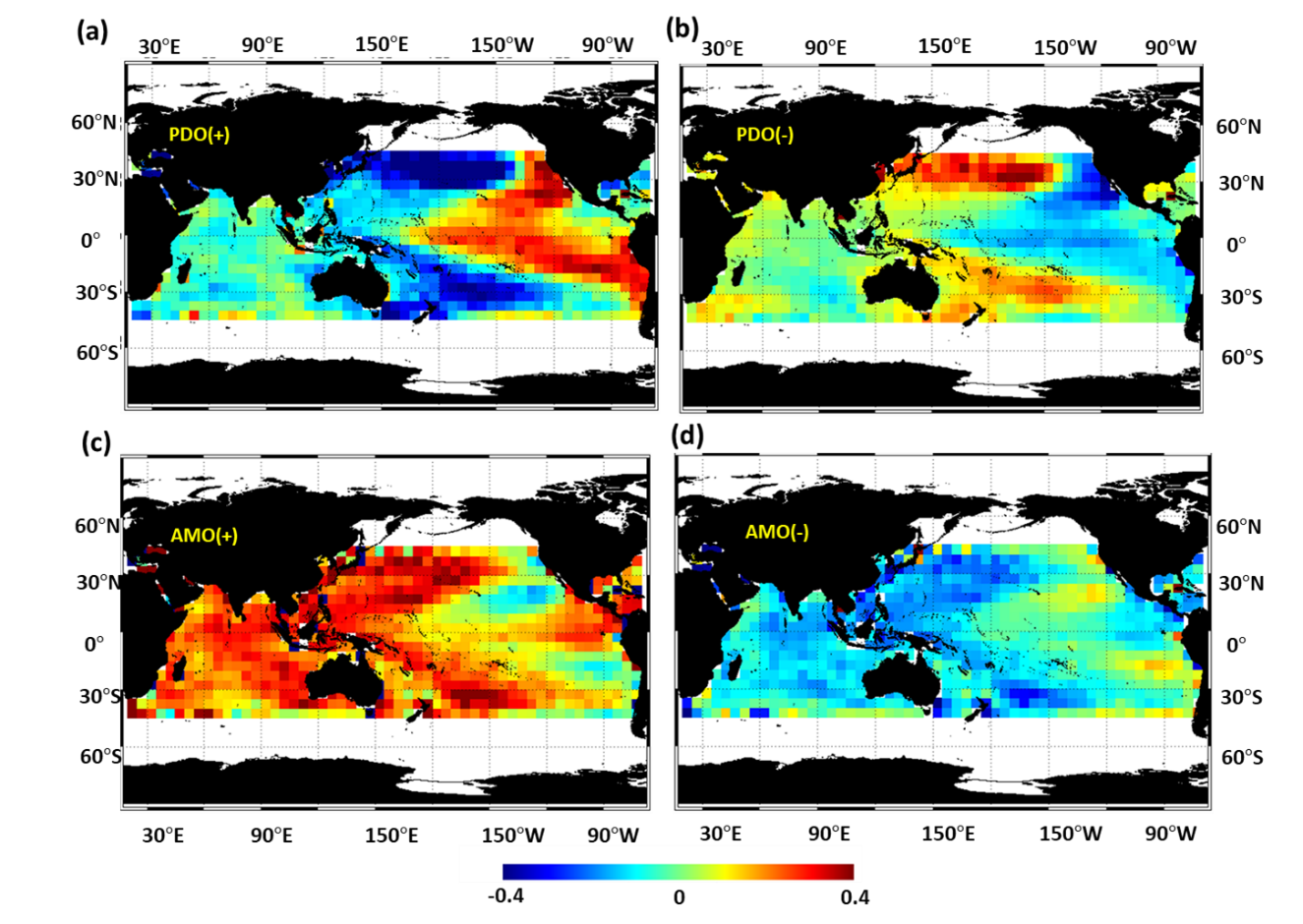


Fig. S2 Spatial distribution of SST anomalies. (a) During PDO positive phases. (b) During PDO negative phases. (c) During AMO positive phases. (d) During AMO negative phases. Figure created with Interactive Data Visualization Solution (IDL v 8.7) software. Software Resource：<https://www.l3harrisgeospatial.com/Software-Technology/IDL>.


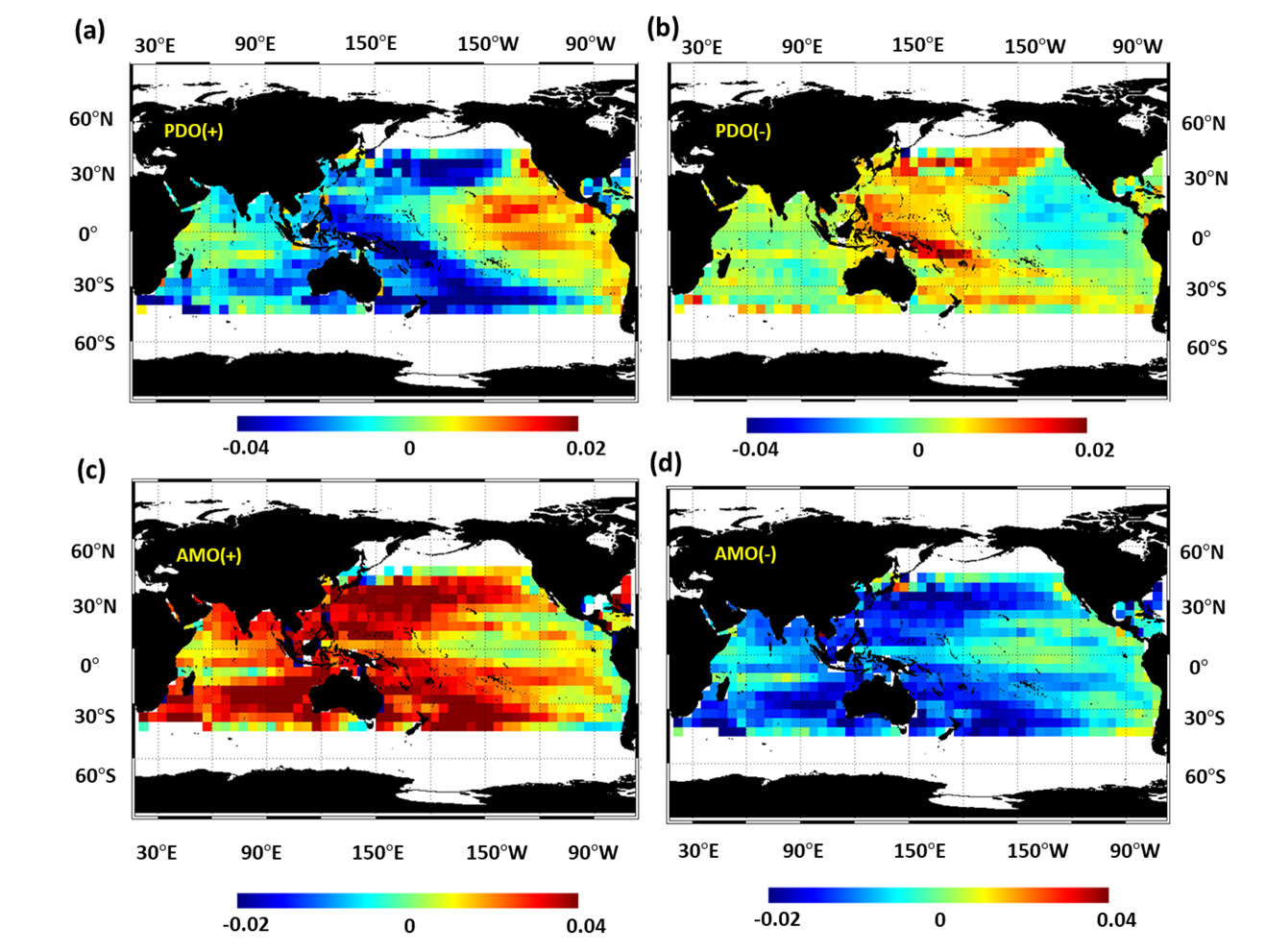


Fig. S3 Spatial distribution of SSH anomalies. (a) During PDO positive phases. (b) During PDO negative phases. (c) During AMO positive phases. (d) During AMO negative phases. Figure created with Interactive Data Visualization Solution (IDL v 8.7) software. Software Resource：<https://www.l3harrisgeospatial.com/Software-Technology/IDL>.


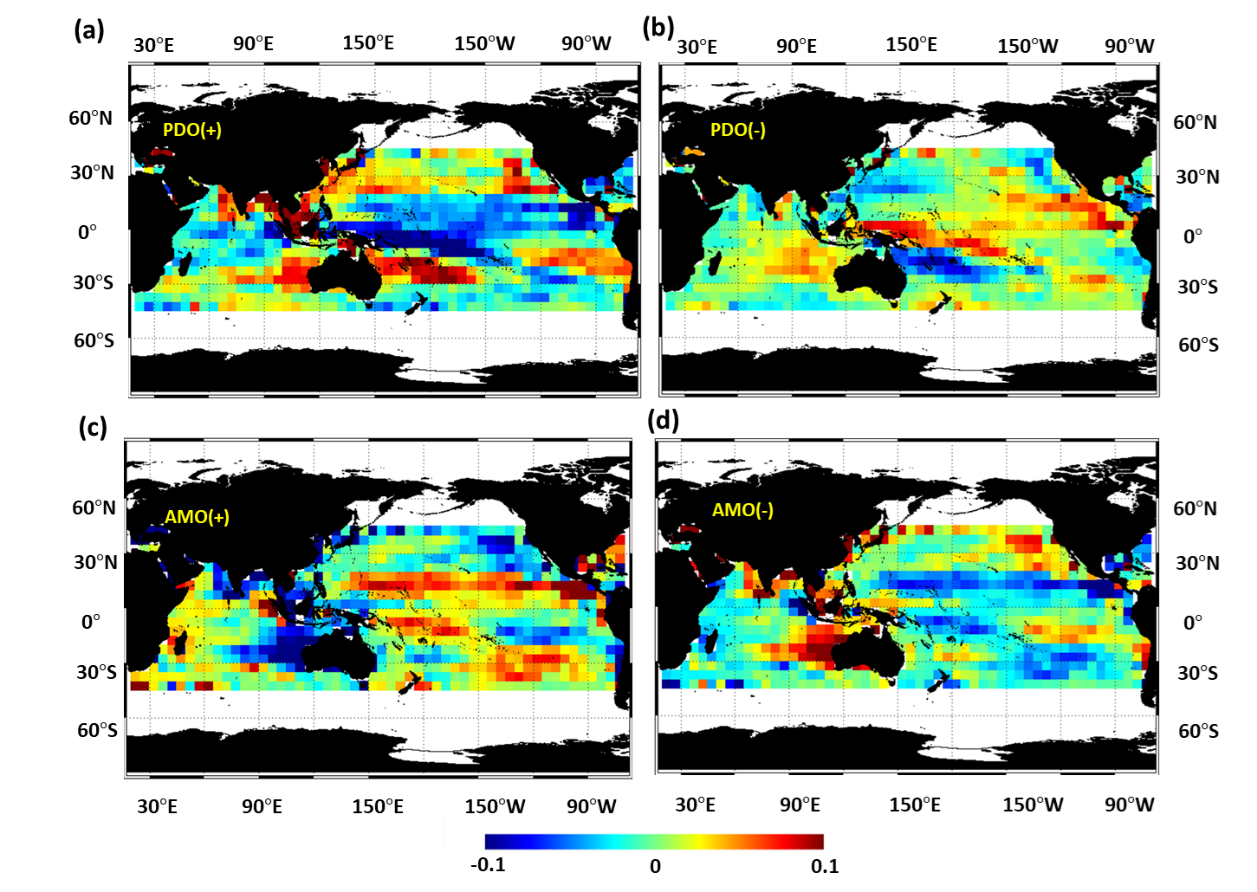


Fig. S4 Spatial distribution of SSS anomalies. (a) During PDO positive phases. (b) During PDO negative phases. (c) During AMO positive phases. (d) During AMO negative phases. Figure created with Interactive Data Visualization Solution (IDL v 8.7) software. Software Resource：<https://www.l3harrisgeospatial.com/Software-Technology/IDL>.


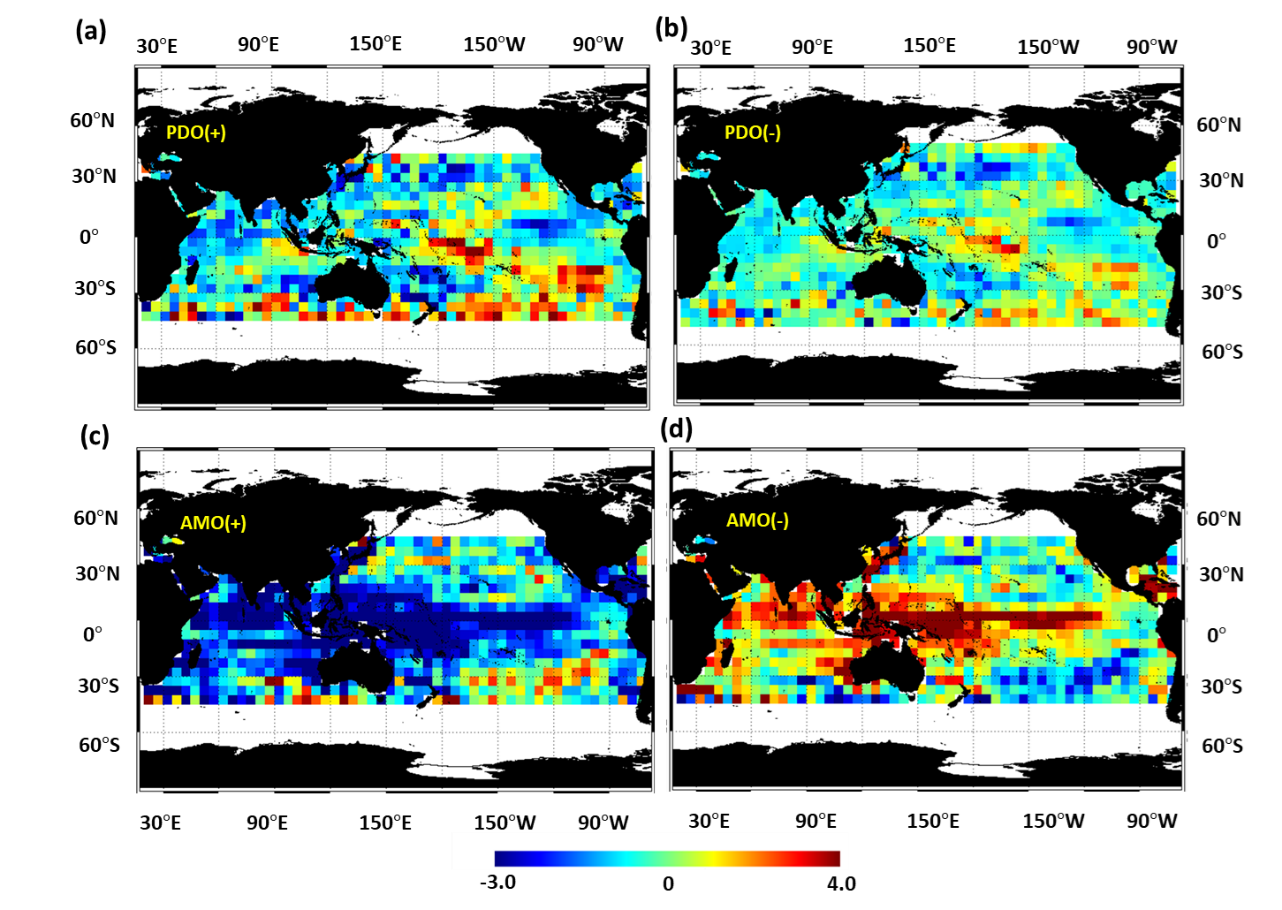


Fig. S5 Spatial distribution of MLD anomalies. (a) During PDO positive phases. (b) During PDO negative phases. (c) During AMO positive phases. (d) During AMO negative phases. Figure created with Interactive Data Visualization Solution (IDL v 8.7) software. Software Resource：<https://www.l3harrisgeospatial.com/Software-Technology/IDL>.


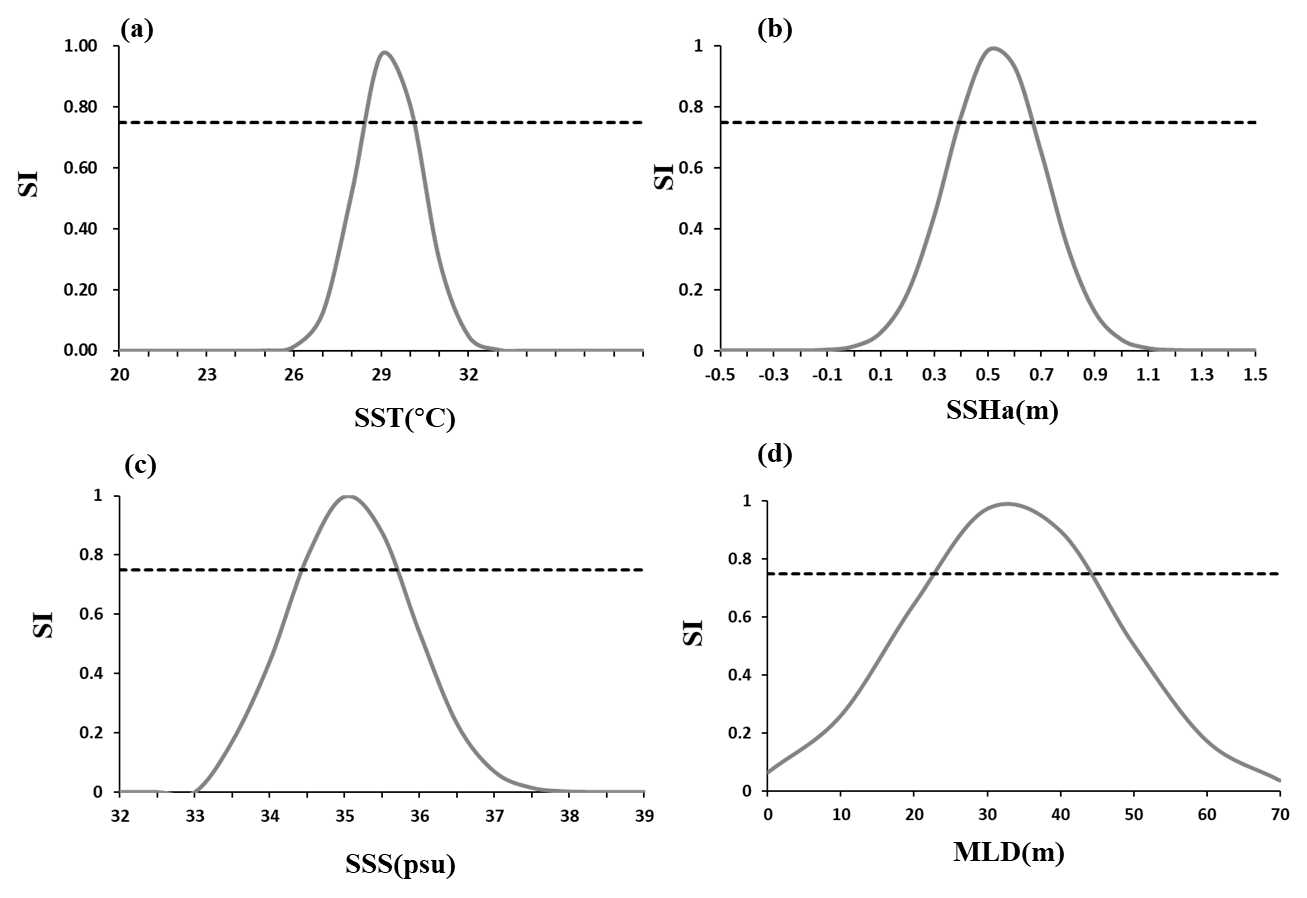


Fig. S6 Suitability index (SI) curves of **(a)** sea surface temperature, **(b)** sea surface height anomalies, **(c)** sea surface salinity, and **(d)** mix layer depth.


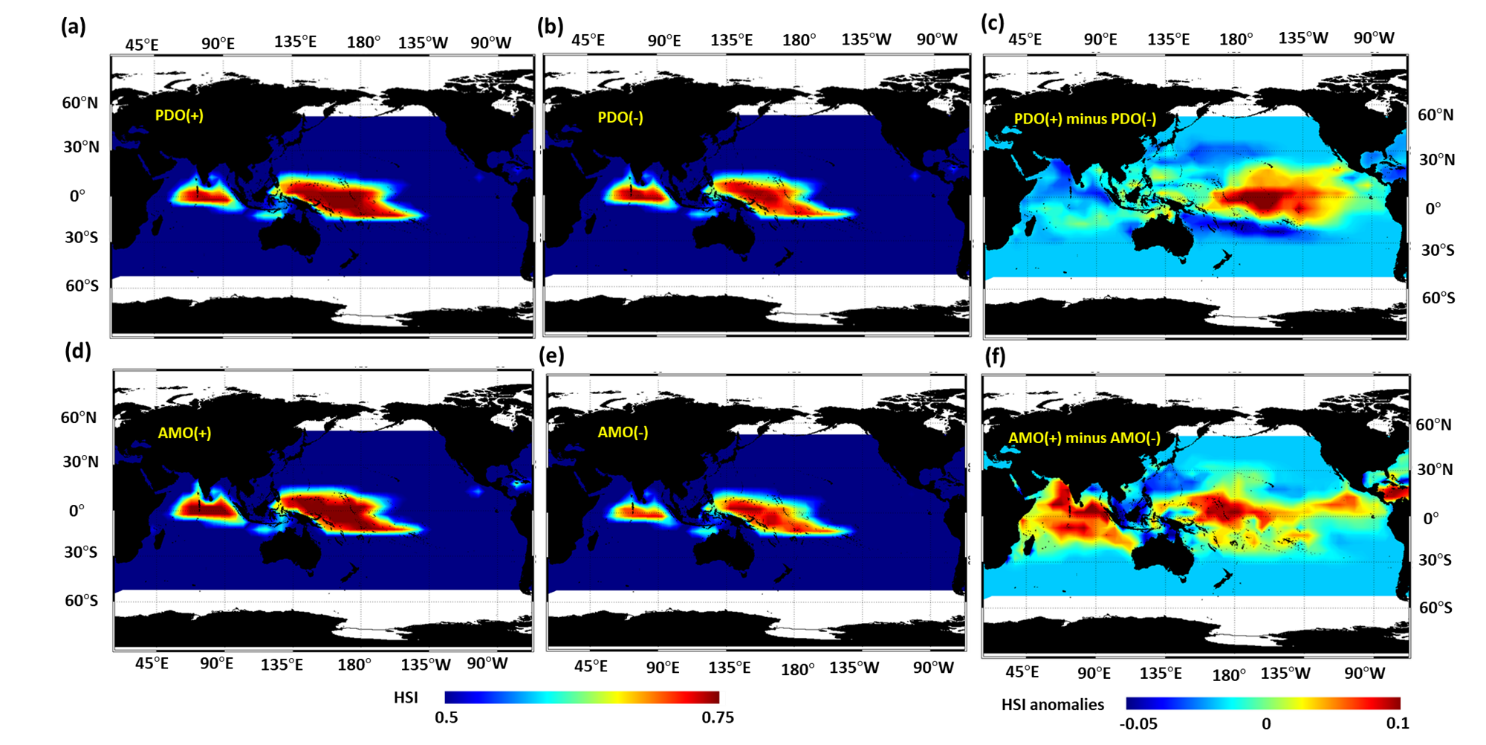


Fig. S7 Yellowfin tuna HSI spatial distributions. (a) During PDO positive phases. (b) During PDO negative phases. (c) During AMO positive phases. (d) During AMO negative phases. (e) Index variation during positive PDO phase minus negative PDO phase. (f) Index variation during positive AMO phase minus negative AMO phase. Figure created with Interactive Data Visualization Solution (IDL v 8.7) software. Software Resource：<https://www.l3harrisgeospatial.com/Software-Technology/IDL>.
