# Supplementary material for: Effects of decadal climate variability on spatiotemporal distribution of Indo-Pacific yellowfin tuna population
Source: Sci Rep. 2022 Aug 12;12:13715. doi: 10.1038/s41598-022-17882-w (PMC9374684; doi:10.1038/s41598-022-17882-w)
Supplement: Supplementary file 2 — Supplementary Information 2. [file 41598_2022_17882_MOESM2_ESM.docx]

| **Year** | **Month** | **Eastern Pacific Ocean standardized CPUE** | **Western Pacific Ocean standardized CPUE** | **Eastern Indian Ocean standardized CPUE** | **Western Indian Ocean standardized CPUE** |
| --- | --- | --- | --- | --- | --- |
| 1971 | 1 | 1.298981 | 3.119273 | 3.050106 | 2.974166 |
| 1971 | 2 | 1.449420 | 3.339773 | 3.214575 | 2.918954 |
| 1971 | 3 | 1.303452 | 3.430869 | 2.735109 | 3.093428 |
| 1971 | 4 | 1.137139 | 3.870443 | 2.930804 | 3.526122 |
| 1971 | 5 | 1.212046 | 3.860706 | 2.380305 | 4.317634 |
| 1971 | 6 | 1.202543 | 4.429853 | 2.651103 | 4.663513 |
| 1971 | 7 | 1.160850 | 3.847368 | 2.003761 | 3.834856 |
| 1971 | 8 | 1.159746 | 4.151404 | 2.187362 | 3.801052 |
| 1971 | 9 | 1.339852 | 3.773611 | 1.733800 | 2.491840 |
| 1971 | 10 | 1.464509 | 3.398018 | 2.091764 | 3.743350 |
| 1971 | 11 | 1.339476 | 3.161974 | 2.689479 | 4.100389 |
| 1971 | 12 | 1.398856 | 3.466580 | 2.732095 | 3.581379 |
| 1972 | 1 | 1.233917 | 3.569179 | 4.242878 | 3.978691 |
| 1972 | 2 | 1.282329 | 4.316932 | 4.873642 | 4.488546 |
| 1972 | 3 | 1.280241 | 4.354079 | 4.217389 | 4.363145 |
| 1972 | 4 | 1.302237 | 4.351480 | 4.088132 | 3.886754 |
| 1972 | 5 | 1.260361 | 4.879518 | 2.885705 | 4.620065 |
| 1972 | 6 | 1.290554 | 4.561654 | 2.433546 | 3.990548 |
| 1972 | 7 | 1.190155 | 4.217274 | 2.277298 | 2.681561 |
| 1972 | 8 | 1.254178 | 3.833521 | 2.128762 | 2.905796 |
| 1972 | 9 | 1.272191 | 4.942761 | 2.051974 | 3.462156 |
| 1972 | 10 | 1.355343 | 4.455514 | 1.736486 | 2.971818 |
| 1972 | 11 | 1.431634 | 3.182723 | 2.135457 | 3.574272 |
| 1972 | 12 | 1.461283 | 2.894041 | 3.053309 | 3.673702 |
| 1973 | 1 | 1.412747 | 4.193638 | 3.745265 | 4.372198 |
| 1973 | 2 | 1.490710 | 3.567449 | 3.986435 | 3.703481 |
| 1973 | 3 | 1.488197 | 3.740107 | 3.521278 | 1.819547 |
| 1973 | 4 | 1.520594 | 4.156673 | 3.084343 | 3.428818 |
| 1973 | 5 | 1.418141 | 3.482390 | 2.001999 | 3.812461 |
| 1973 | 6 | 1.126520 | 3.798550 | 1.764141 | 3.675376 |
| 1973 | 7 | 1.310264 | 3.601281 | 1.781829 | 2.835559 |
| 1973 | 8 | 1.427499 | 3.508203 | 1.469962 | 3.690868 |
| 1973 | 9 | 1.297187 | 3.299336 | 1.732613 | 2.404455 |
| 1973 | 10 | 1.274697 | 3.272813 | 1.489302 | 2.496466 |
| 1973 | 11 | 1.260616 | 2.735371 | 1.771194 | 2.333881 |
| 1973 | 12 | 1.386628 | 2.667623 | 1.830001 | 3.088394 |
| 1974 | 1 | 1.178898 | 3.098334 | 2.925491 | 3.329071 |
| 1974 | 2 | 1.368185 | 3.298888 | 3.335043 | 3.652410 |
| 1974 | 3 | 1.423820 | 3.725621 | 2.644775 | 3.041491 |
| 1974 | 4 | 1.373778 | 3.925000 | 1.947322 | 3.014226 |
| 1974 | 5 | 1.206183 | 3.704273 | 2.179340 | 3.027318 |
| 1974 | 6 | 1.175892 | 3.205327 | 1.847395 | 2.943534 |
| 1974 | 7 | 1.300989 | 3.428287 | 1.983008 | 3.685438 |
| 1974 | 8 | 1.258464 | 3.995216 | 1.863430 | 3.098819 |
| 1974 | 9 | 1.188490 | 4.729659 | 1.845205 | 2.598679 |
| 1974 | 10 | 1.301723 | 4.333516 | 1.652517 | 2.611816 |
| 1974 | 11 | 1.232861 | 3.309892 | 1.930188 | 2.304602 |
| 1974 | 12 | 1.426090 | 3.255490 | 2.084100 | 2.331644 |
| 1975 | 1 | 1.165068 | 3.644078 | 3.026418 | 3.204253 |
| 1975 | 2 | 1.297531 | 3.993414 | 2.993263 | 3.979380 |
| 1975 | 3 | 1.543361 | 4.083297 | 2.984880 | 4.236123 |
| 1975 | 4 | 1.332857 | 3.874321 | 2.829360 | 4.567059 |
| 1975 | 5 | 1.263759 | 3.991216 | 2.607878 | 3.690140 |
| 1975 | 6 | 1.190969 | 3.783255 | 2.151109 | 3.721242 |
| 1975 | 7 | 1.173713 | 3.950022 | 2.025964 | 2.399066 |
| 1975 | 8 | 1.381159 | 3.980970 | 1.986313 | 2.659577 |
| 1975 | 9 | 1.465193 | 3.811288 | 1.637553 | 2.864435 |
| 1975 | 10 | 1.557338 | 4.053285 | 1.511101 | 3.865309 |
| 1975 | 11 | 1.522898 | 3.835468 | 1.627986 | 3.443478 |
| 1975 | 12 | 1.542911 | 3.119529 | 2.031099 | 3.365006 |
| 1976 | 1 | 1.395339 | 3.967996 | 3.829380 | 2.554454 |
| 1976 | 2 | 1.412765 | 3.853852 | 3.556536 | 2.128487 |
| 1976 | 3 | 1.326241 | 3.806346 | 3.022998 | 2.364242 |
| 1976 | 4 | 1.274113 | 4.416593 | 2.920457 | 2.977776 |
| 1976 | 5 | 1.267582 | 4.512448 | 2.307147 | 3.352973 |
| 1976 | 6 | 1.242306 | 4.331835 | 1.668009 | 3.652246 |
| 1976 | 7 | 1.299213 | 4.061254 | 1.673566 | 2.525905 |
| 1976 | 8 | 1.286947 | 3.990150 | 1.727043 | 2.501076 |
| 1976 | 9 | 1.549417 | 3.566713 | 1.391687 | 2.362829 |
| 1976 | 10 | 1.422108 | 3.874355 | 1.481701 | 2.569569 |
| 1976 | 11 | 1.447760 | 3.445009 | 1.472500 | 2.624876 |
| 1976 | 12 | 1.638780 | 3.746181 | 1.960417 | 2.693089 |
| 1977 | 1 | 1.524866 | 3.369745 | 2.247442 | 3.777037 |
| 1977 | 2 | 1.497733 | 4.162966 | 2.148770 | 4.445221 |
| 1977 | 3 | 1.581097 | 4.366117 | 2.692066 | 4.537847 |
| 1977 | 4 | 1.620470 | 4.691216 | 2.495779 | 4.179213 |
| 1977 | 5 | 1.565785 | 4.834539 | 2.337730 | 4.932730 |
| 1977 | 6 | 1.426660 | 4.151150 | 1.933780 | 4.728135 |
| 1977 | 7 | 1.464079 | 4.173352 | 2.103937 | 3.875785 |
| 1977 | 8 | 1.537414 | 4.406020 | 1.838419 | 3.703164 |
| 1977 | 9 | 1.415839 | 4.059456 | 1.583430 | 3.729589 |
| 1977 | 10 | 1.395494 | 3.640664 | 1.411927 | 3.834416 |
| 1977 | 11 | 1.458478 | 4.153370 | 1.733076 | 4.137197 |
| 1977 | 12 | 1.627069 | 3.947940 | 2.286194 | 4.427144 |
| 1978 | 1 | 1.456383 | 3.412262 | 2.327547 | 4.118331 |
| 1978 | 2 | 1.469739 | 3.931679 | 2.871981 | 4.702645 |
| 1978 | 3 | 1.482561 | 4.083828 | 2.789990 | 4.907363 |
| 1978 | 4 | 1.422020 | 3.776111 | 2.834092 | 4.730921 |
| 1978 | 5 | 1.415581 | 3.659297 | 2.099149 | 4.275496 |
| 1978 | 6 | 1.249190 | 3.213987 | 2.377340 | 4.405856 |
| 1978 | 7 | 1.295796 | 3.226290 | 2.180969 | 3.137709 |
| 1978 | 8 | 1.448411 | 3.531217 | 1.957216 | 2.754249 |
| 1978 | 9 | 1.356200 | 3.909352 | 1.886398 | 3.424943 |
| 1978 | 10 | 1.451834 | 3.598227 | 1.685276 | 3.753899 |
| 1978 | 11 | 1.486101 | 3.513549 | 2.200345 | 4.016599 |
| 1978 | 12 | 1.498156 | 3.716334 | 2.259373 | 4.183253 |
| 1979 | 1 | 1.423544 | 3.473288 | 2.396780 | 3.778240 |
| 1979 | 2 | 1.382964 | 3.286317 | 2.686404 | 3.581538 |
| 1979 | 3 | 1.445584 | 3.768637 | 2.351348 | 3.188112 |
| 1979 | 4 | 1.411660 | 3.825285 | 2.985075 | 3.641868 |
| 1979 | 5 | 1.269095 | 4.169878 | 2.765529 | 3.955620 |
| 1979 | 6 | 1.357224 | 4.209229 | 2.436616 | 3.493733 |
| 1979 | 7 | 1.392965 | 4.373102 | 2.152667 | 3.611578 |
| 1979 | 8 | 1.496490 | 4.363226 | 1.399264 | 2.795824 |
| 1979 | 9 | 1.361206 | 4.075245 | 1.519521 | 2.707938 |
| 1979 | 10 | 1.382981 | 3.716215 | 1.439863 | 2.878211 |
| 1979 | 11 | 1.534443 | 3.788694 | 1.765961 | 4.016139 |
| 1979 | 12 | 1.448035 | 3.395577 | 1.823341 | 3.302231 |
| 1980 | 1 | 1.373843 | 3.962691 | 2.019989 | 3.276367 |
| 1980 | 2 | 1.417038 | 4.149397 | 2.104680 | 3.186584 |
| 1980 | 3 | 1.668253 | 3.841189 | 2.340513 | 2.670227 |
| 1980 | 4 | 1.509688 | 4.047093 | 2.419948 | 3.035309 |
| 1980 | 5 | 1.251501 | 3.884163 | 2.205689 | 2.735047 |
| 1980 | 6 | 1.305159 | 3.991007 | 2.003886 | 2.726591 |
| 1980 | 7 | 1.294608 | 3.367369 | 1.991614 | 2.222788 |
| 1980 | 8 | 1.313610 | 3.804172 | 1.806285 | 2.641695 |
| 1980 | 9 | 1.370191 | 3.555800 | 1.426260 | 2.490074 |
| 1980 | 10 | 1.262928 | 3.172019 | 1.520850 | 3.426248 |
| 1980 | 11 | 1.210860 | 2.772463 | 1.662105 | 3.534504 |
| 1980 | 12 | 1.139628 | 2.554254 | 1.788875 | 3.804516 |
| 1981 | 1 | 1.096684 | 2.780653 | 2.143105 | 2.945589 |
| 1981 | 2 | 1.138071 | 2.784231 | 2.267243 | 2.955767 |
| 1981 | 3 | 1.186421 | 2.880596 | 2.327741 | 2.602562 |
| 1981 | 4 | 1.225086 | 3.370536 | 2.259026 | 2.793871 |
| 1981 | 5 | 1.201748 | 2.909562 | 2.263994 | 2.513982 |
| 1981 | 6 | 1.172829 | 2.777559 | 1.785707 | 2.868253 |
| 1981 | 7 | 1.042843 | 2.676401 | 1.943952 | 2.695541 |
| 1981 | 8 | 0.991594 | 2.806184 | 1.802501 | 2.854437 |
| 1981 | 9 | 1.129058 | 2.820706 | 1.373115 | 2.495867 |
| 1981 | 10 | 1.087710 | 2.430617 | 1.473345 | 2.721079 |
| 1981 | 11 | 1.244887 | 2.445080 | 1.832438 | 3.656412 |
| 1981 | 12 | 1.257050 | 2.426880 | 1.414485 | 3.063395 |
| 1982 | 1 | 1.109750 | 2.551748 | 1.955013 | 3.273082 |
| 1982 | 2 | 1.194757 | 2.924460 | 2.253598 | 2.818436 |
| 1982 | 3 | 1.210500 | 3.146294 | 2.504009 | 2.413448 |
| 1982 | 4 | 1.250500 | 3.265725 | 2.222255 | 2.861088 |
| 1982 | 5 | 1.194056 | 3.204688 | 2.197982 | 2.944301 |
| 1982 | 6 | 1.100913 | 3.189849 | 1.884701 | 3.324590 |
| 1982 | 7 | 1.248823 | 3.045021 | 1.661727 | 2.493452 |
| 1982 | 8 | 1.280427 | 3.382990 | 1.609805 | 2.395380 |
| 1982 | 9 | 1.237725 | 3.018932 | 1.402080 | 2.580711 |
| 1982 | 10 | 1.286690 | 3.353972 | 1.509916 | 3.181090 |
| 1982 | 11 | 1.202095 | 2.992268 | 1.810010 | 3.698333 |
| 1982 | 12 | 1.228021 | 3.577230 | 1.844873 | 3.567182 |
| 1983 | 1 | 1.165688 | 3.105218 | 2.225249 | 2.662718 |
| 1983 | 2 | 1.280396 | 3.373583 | 2.410868 | 3.253719 |
| 1983 | 3 | 1.326854 | 3.036187 | 2.595368 | 2.907370 |
| 1983 | 4 | 1.130969 | 3.434911 | 2.080474 | 3.454617 |
| 1983 | 5 | 1.119796 | 3.247696 | 1.701035 | 3.220815 |
| 1983 | 6 | 1.144579 | 3.612883 | 1.484007 | 2.941630 |
| 1983 | 7 | 1.252046 | 3.235091 | 1.550592 | 2.146841 |
| 1983 | 8 | 1.289605 | 3.465992 | 1.730375 | 2.061714 |
| 1983 | 9 | 1.308315 | 3.292031 | 1.414456 | 2.205741 |
| 1983 | 10 | 1.287534 | 2.730153 | 1.444642 | 2.571320 |
| 1983 | 11 | 1.229650 | 3.109213 | 1.556960 | 3.099673 |
| 1983 | 12 | 1.377304 | 3.509858 | 1.509543 | 2.795483 |
| 1984 | 1 | 1.143731 | 3.167685 | 1.806412 | 3.337653 |
| 1984 | 2 | 1.175232 | 3.596562 | 2.020760 | 2.858744 |
| 1984 | 3 | 1.250679 | 3.252513 | 1.920620 | 2.621921 |
| 1984 | 4 | 1.244313 | 3.491992 | 1.593166 | 2.852915 |
| 1984 | 5 | 1.139014 | 3.299408 | 1.540095 | 2.723661 |
| 1984 | 6 | 1.184757 | 3.222007 | 1.628994 | 3.168000 |
| 1984 | 7 | 1.204901 | 3.496619 | 1.563485 | 2.413829 |
| 1984 | 8 | 1.213449 | 3.566352 | 1.532524 | 2.576434 |
| 1984 | 9 | 1.199207 | 3.151222 | 1.366136 | 2.342787 |
| 1984 | 10 | 1.221232 | 3.100857 | 1.317808 | 2.591212 |
| 1984 | 11 | 1.405609 | 3.116342 | 1.296969 | 2.879837 |
| 1984 | 12 | 1.384867 | 3.164451 | 1.567662 | 2.764380 |
| 1985 | 1 | 1.383913 | 3.339741 | 1.866426 | 2.529274 |
| 1985 | 2 | 1.474293 | 3.494197 | 2.132326 | 2.514099 |
| 1985 | 3 | 1.516216 | 3.235918 | 2.423943 | 2.496812 |
| 1985 | 4 | 1.644555 | 3.806606 | 2.282610 | 2.890114 |
| 1985 | 5 | 1.688590 | 3.998035 | 1.682317 | 2.724555 |
| 1985 | 6 | 1.489551 | 3.568432 | 1.803907 | 2.525291 |
| 1985 | 7 | 1.643904 | 3.616835 | 1.756903 | 2.466715 |
| 1985 | 8 | 1.660795 | 4.026528 | 1.426108 | 3.191254 |
| 1985 | 9 | 1.540002 | 3.269278 | 1.303837 | 2.708106 |
| 1985 | 10 | 1.477724 | 3.231804 | 1.195784 | 2.815998 |
| 1985 | 11 | 1.579972 | 3.389495 | 1.431746 | 3.124826 |
| 1985 | 12 | 1.537949 | 3.409343 | 1.680873 | 3.273576 |
| 1986 | 1 | 1.406412 | 2.962710 | 2.065015 | 3.080317 |
| 1986 | 2 | 1.392255 | 3.299365 | 2.407440 | 2.412721 |
| 1986 | 3 | 1.545302 | 3.098912 | 2.211418 | 2.350440 |
| 1986 | 4 | 1.412427 | 3.425161 | 2.328321 | 2.940076 |
| 1986 | 5 | 1.427215 | 3.852318 | 1.735002 | 3.438807 |
| 1986 | 6 | 1.442654 | 3.457177 | 1.634686 | 2.856240 |
| 1986 | 7 | 1.464131 | 3.336979 | 1.304080 | 2.377347 |
| 1986 | 8 | 1.545909 | 3.293452 | 1.418815 | 2.883019 |
| 1986 | 9 | 1.467270 | 3.149619 | 1.307235 | 3.137351 |
| 1986 | 10 | 1.361786 | 2.888662 | 1.408980 | 2.767525 |
| 1986 | 11 | 1.392350 | 3.156676 | 1.680087 | 3.467335 |
| 1986 | 12 | 1.404089 | 3.488723 | 1.713780 | 3.788908 |
| 1987 | 1 | 1.201733 | 3.346471 | 2.271330 | 2.981527 |
| 1987 | 2 | 1.210716 | 3.739737 | 2.336233 | 3.113188 |
| 1987 | 3 | 1.312576 | 3.622270 | 2.344095 | 2.704177 |
| 1987 | 4 | 1.132994 | 4.053694 | 2.137938 | 3.158569 |
| 1987 | 5 | 1.115850 | 3.987001 | 1.825914 | 3.391396 |
| 1987 | 6 | 1.216352 | 3.812183 | 1.415644 | 3.134072 |
| 1987 | 7 | 1.124774 | 3.645953 | 1.294205 | 2.890048 |
| 1987 | 8 | 1.286224 | 4.169791 | 1.201244 | 2.967604 |
| 1987 | 9 | 1.214975 | 3.583721 | 1.121950 | 2.072822 |
| 1987 | 10 | 1.068974 | 3.313552 | 1.253336 | 2.636993 |
| 1987 | 11 | 1.096370 | 3.042682 | 1.437243 | 3.124772 |
| 1987 | 12 | 1.105429 | 3.429658 | 1.592797 | 3.020084 |
| 1988 | 1 | 1.078130 | 2.979368 | 2.340067 | 2.487180 |
| 1988 | 2 | 1.173784 | 3.132051 | 2.144752 | 3.367922 |
| 1988 | 3 | 1.149239 | 2.975307 | 2.064612 | 2.331386 |
| 1988 | 4 | 1.135989 | 3.378917 | 2.179834 | 1.969641 |
| 1988 | 5 | 0.960072 | 3.213419 | 1.000659 | 2.162102 |
| 1988 | 6 | 0.967721 | 2.975387 | 1.027150 | 1.708122 |
| 1988 | 7 | 0.905197 | 2.827786 | 1.081613 | 1.518726 |
| 1988 | 8 | 0.963886 | 3.030663 | 1.123205 | 1.529066 |
| 1988 | 9 | 0.875416 | 2.615534 | 1.013740 | 1.671588 |
| 1988 | 10 | 0.905251 | 2.504858 | 1.142466 | 2.122509 |
| 1988 | 11 | 0.987054 | 2.613700 | 1.246426 | 2.419957 |
| 1988 | 12 | 1.105047 | 2.792282 | 1.582442 | 3.124944 |
| 1989 | 1 | 1.010381 | 2.971465 | 1.896650 | 3.137333 |
| 1989 | 2 | 1.162299 | 3.058545 | 2.084169 | 2.724789 |
| 1989 | 3 | 1.127264 | 2.902218 | 2.123595 | 2.313236 |
| 1989 | 4 | 0.997795 | 3.529512 | 2.149513 | 2.453656 |
| 1989 | 5 | 0.959860 | 3.559890 | 1.616740 | 2.358587 |
| 1989 | 6 | 1.061044 | 3.318785 | 0.995917 | 2.329746 |
| 1989 | 7 | 1.218075 | 3.345576 | 1.286614 | 1.591531 |
| 1989 | 8 | 1.069569 | 3.267778 | 1.091864 | 1.534241 |
| 1989 | 9 | 1.204237 | 3.157753 | 1.036332 | 2.040754 |
| 1989 | 10 | 1.099225 | 3.588316 | 1.339369 | 1.929392 |
| 1989 | 11 | 1.206818 | 3.976900 | 1.342135 | 2.366065 |
| 1989 | 12 | 1.181196 | 4.056632 | 1.321986 | 3.338395 |
| 1990 | 1 | 1.093090 | 3.579972 | 2.083716 | 2.771935 |
| 1990 | 2 | 1.192876 | 3.452937 | 1.983379 | 2.550643 |
| 1990 | 3 | 1.329458 | 3.793238 | 1.939076 | 2.550829 |
| 1990 | 4 | 1.305241 | 4.345890 | 3.325910 | 3.237917 |
| 1990 | 5 | 1.390930 | 3.534255 | 0.980980 | 2.634730 |
| 1990 | 6 | 1.260639 | 3.751391 | 1.149093 | 2.550737 |
| 1990 | 7 | 1.405596 | 4.515444 | 0.914808 | 2.101086 |
| 1990 | 8 | 1.382482 | 4.550874 | 0.987282 | 1.733781 |
| 1990 | 9 | 1.525867 | 3.993083 | 1.271159 | 2.082493 |
| 1990 | 10 | 1.463559 | 4.238630 | 1.207321 | 2.468465 |
| 1990 | 11 | 1.357312 | 3.741092 | 1.426292 | 2.784868 |
| 1990 | 12 | 1.474223 | 3.596836 | 1.190201 | 2.160529 |
| 1991 | 1 | 1.270011 | 3.472507 | 1.862555 | 2.047704 |
| 1991 | 2 | 1.247841 | 3.499319 | 1.956983 | 1.671131 |
| 1991 | 3 | 1.227234 | 2.763769 | 1.828920 | 1.970242 |
| 1991 | 4 | 1.210315 | 3.146644 | 1.311102 | 2.445957 |
| 1991 | 5 | 1.209435 | 3.082017 | 1.186370 | 2.275127 |
| 1991 | 6 | 1.245770 | 2.974072 | 1.264132 | 1.687230 |
| 1991 | 7 | 1.257733 | 3.406170 | 1.097358 | 1.380122 |
| 1991 | 8 | 1.221291 | 3.480570 | 0.899028 | 1.963787 |
| 1991 | 9 | 1.310813 | 2.976639 | 0.758977 | 2.067315 |
| 1991 | 10 | 1.232613 | 3.055553 | 0.905730 | 2.068347 |
| 1991 | 11 | 1.154063 | 2.770327 | 0.866653 | 1.977849 |
| 1991 | 12 | 1.262399 | 3.086499 | 0.941507 | 2.225863 |
| 1992 | 1 | 1.094005 | 3.585949 | 1.630557 | 2.908753 |
| 1992 | 2 | 1.311063 | 3.594171 | 1.787528 | 2.487176 |
| 1992 | 3 | 1.079146 | 3.812560 | 1.256478 | 2.341036 |
| 1992 | 4 | 1.145657 | 3.782166 | 0.940910 | 1.856071 |
| 1992 | 5 | 1.157413 | 3.269008 | 1.272776 | 2.255084 |
| 1992 | 6 | 1.135949 | 3.367273 | 1.089483 | 2.594819 |
| 1992 | 7 | 0.979475 | 3.583725 | 1.219511 | 1.829566 |
| 1992 | 8 | 1.089617 | 3.746476 | 0.803315 | 2.634980 |
| 1992 | 9 | 1.038300 | 3.058113 | 0.676287 | 2.758991 |
| 1992 | 10 | 0.958442 | 3.298724 | 1.044032 | 3.657948 |
| 1992 | 11 | 1.007017 | 3.787353 | 1.161393 | 4.307007 |
| 1992 | 12 | 1.097243 | 3.352003 | 1.033390 | 3.856283 |
| 1993 | 1 | 1.162018 | 3.183404 | 1.847825 | 3.015068 |
| 1993 | 2 | 1.129624 | 2.987748 | 1.826366 | 3.452724 |
| 1993 | 3 | 0.995073 | 2.883974 | 1.347694 | 2.861198 |
| 1993 | 4 | 1.123921 | 3.246184 | 0.859998 | 2.430875 |
| 1993 | 5 | 1.067624 | 3.446175 | 1.375242 | 2.595715 |
| 1993 | 6 | 1.115004 | 3.187621 | 1.369410 | 2.309041 |
| 1993 | 7 | 1.147758 | 3.214341 | 1.366771 | 2.275067 |
| 1993 | 8 | 1.113914 | 3.511358 | 1.152408 | 2.138030 |
| 1993 | 9 | 1.085216 | 3.363669 | 0.951419 | 2.333906 |
| 1993 | 10 | 0.947132 | 3.300195 | 1.091364 | 2.455588 |
| 1993 | 11 | 0.860227 | 2.801595 | 1.118103 | 2.695375 |
| 1993 | 12 | 1.037775 | 2.845185 | 1.080248 | 2.225619 |
| 1994 | 1 | 1.071751 | 2.824059 | 1.252994 | 2.221546 |
| 1994 | 2 | 1.112405 | 3.002913 | 1.516613 | 2.327410 |
| 1994 | 3 | 1.054409 | 2.860727 | 1.275147 | 2.030329 |
| 1994 | 4 | 1.055990 | 3.388410 | 1.001399 | 1.950967 |
| 1994 | 5 | 0.870191 | 3.334248 | 0.936306 | 1.831808 |
| 1994 | 6 | 0.951231 | 3.575743 | 1.204029 | 1.736340 |
| 1994 | 7 | 0.981187 | 3.354963 | 0.906349 | 1.724056 |
| 1994 | 8 | 1.005516 | 3.539737 | 0.816539 | 1.403076 |
| 1994 | 9 | 1.030393 | 3.521894 | 0.806914 | 1.500008 |
| 1994 | 10 | 1.002075 | 3.248826 | 0.834205 | 1.977892 |
| 1994 | 11 | 1.079441 | 3.056746 | 0.860341 | 1.836148 |
| 1994 | 12 | 1.142124 | 2.729695 | 1.252950 | 1.500230 |
| 1995 | 1 | 1.126235 | 3.516209 | 1.481794 | 1.515166 |
| 1995 | 2 | 1.107457 | 2.982743 | 1.461031 | 1.628674 |
| 1995 | 3 | 0.982933 | 2.778787 | 1.314483 | 1.872745 |
| 1995 | 4 | 0.942430 | 3.068432 | 1.039708 | 1.953571 |
| 1995 | 5 | 0.892997 | 2.882684 | 0.808314 | 1.562439 |
| 1995 | 6 | 0.994010 | 2.691361 | 0.831977 | 1.709258 |
| 1995 | 7 | 0.978814 | 2.789349 | 0.806552 | 1.433865 |
| 1995 | 8 | 1.026913 | 2.813033 | 0.849211 | 1.264083 |
| 1995 | 9 | 0.944045 | 2.610550 | 0.831781 | 1.600490 |
| 1995 | 10 | 0.894758 | 2.517064 | 0.951797 | 1.815754 |
| 1995 | 11 | 0.930349 | 2.221554 | 1.068614 | 1.939841 |
| 1995 | 12 | 0.922654 | 2.436397 | 1.143503 | 2.069544 |
| 1996 | 1 | 0.870549 | 2.511112 | 1.304053 | 1.895145 |
| 1996 | 2 | 0.874159 | 2.577070 | 1.461059 | 1.503191 |
| 1996 | 3 | 0.825487 | 2.547447 | 1.154999 | 1.620606 |
| 1996 | 4 | 0.800269 | 2.754960 | 1.044555 | 1.637679 |
| 1996 | 5 | 0.839355 | 2.782361 | 0.738276 | 1.952844 |
| 1996 | 6 | 0.838287 | 2.853631 | 0.829282 | 1.401298 |
| 1996 | 7 | 0.929179 | 2.951342 | 0.883121 | 1.363300 |
| 1996 | 8 | 0.910144 | 3.089765 | 1.035205 | 1.493457 |
| 1996 | 9 | 1.092050 | 2.635950 | 0.769662 | 1.687457 |
| 1996 | 10 | 0.987741 | 2.496189 | 0.829616 | 1.475188 |
| 1996 | 11 | 0.992874 | 2.457551 | 0.922258 | 1.855366 |
| 1996 | 12 | 1.168857 | 2.347613 | 1.029466 | 1.684975 |
| 1997 | 1 | 1.011514 | 2.886498 | 2.144755 | 1.574725 |
| 1997 | 2 | 0.933044 | 2.795968 | 1.547897 | 1.513417 |
| 1997 | 3 | 0.838557 | 2.806754 | 1.453931 | 1.553310 |
| 1997 | 4 | 0.870249 | 3.046147 | 1.123661 | 1.929880 |
| 1997 | 5 | 0.739974 | 2.883385 | 0.800801 | 1.913957 |
| 1997 | 6 | 0.913208 | 3.034856 | 1.105759 | 1.802039 |
| 1997 | 7 | 0.927192 | 2.939286 | 1.007157 | 1.680620 |
| 1997 | 8 | 1.058012 | 3.456333 | 0.865327 | 1.563263 |
| 1997 | 9 | 0.988443 | 3.421492 | 0.814954 | 1.588592 |
| 1997 | 10 | 0.995509 | 3.201192 | 0.967763 | 1.792198 |
| 1997 | 11 | 1.141203 | 3.398947 | 1.047330 | 1.689215 |
| 1997 | 12 | 1.154916 | 3.512282 | 0.993136 | 1.798138 |
| 1998 | 1 | 0.966325 | 3.128816 | 2.032331 | 1.939272 |
| 1998 | 2 | 0.952468 | 2.937713 | 1.851580 | 2.153189 |
| 1998 | 3 | 0.947466 | 2.967617 | 1.325323 | 1.932486 |
| 1998 | 4 | 0.939588 | 2.890241 | 1.204170 | 1.959736 |
| 1998 | 5 | 0.821069 | 2.968914 | 0.916758 | 1.856389 |
| 1998 | 6 | 0.919415 | 2.959604 | 0.889099 | 1.618051 |
| 1998 | 7 | 0.995069 | 3.020174 | 0.797962 | 1.862751 |
| 1998 | 8 | 1.014244 | 3.577795 | 0.675499 | 1.595542 |
| 1998 | 9 | 0.848758 | 3.240774 | 0.677666 | 1.469052 |
| 1998 | 10 | 0.847732 | 2.938244 | 0.876626 | 1.719889 |
| 1998 | 11 | 1.005281 | 2.755673 | 0.948720 | 1.841667 |
| 1998 | 12 | 0.926270 | 3.067329 | 1.060614 | 1.728919 |
| 1999 | 1 | 0.842356 | 2.795676 | 1.517007 | 1.597712 |
| 1999 | 2 | 0.810478 | 2.667886 | 1.534192 | 1.751579 |
| 1999 | 3 | 0.848708 | 2.574313 | 1.150864 | 1.585237 |
| 1999 | 4 | 0.775681 | 2.770259 | 1.053050 | 1.462974 |
| 1999 | 5 | 0.834580 | 2.695133 | 0.943334 | 1.973195 |
| 1999 | 6 | 0.700284 | 2.523569 | 1.142121 | 1.461646 |
| 1999 | 7 | 0.996048 | 2.618524 | 0.866393 | 1.344569 |
| 1999 | 8 | 0.951552 | 2.729890 | 0.770593 | 1.460740 |
| 1999 | 9 | 0.890517 | 2.407574 | 0.765405 | 1.637847 |
| 1999 | 10 | 0.915718 | 2.377852 | 1.012604 | 1.684738 |
| 1999 | 11 | 0.862492 | 2.428039 | 0.987510 | 1.745156 |
| 1999 | 12 | 0.867273 | 2.454418 | 0.934661 | 1.970356 |
| 2000 | 1 | 0.805951 | 2.524303 | 1.694905 | 1.571159 |
| 2000 | 2 | 0.902291 | 2.223562 | 1.350839 | 1.594383 |
| 2000 | 3 | 0.800435 | 2.413357 | 1.133847 | 1.963443 |
| 2000 | 4 | 0.991483 | 2.329289 | 1.077811 | 1.723527 |
| 2000 | 5 | 0.809794 | 2.600688 | 1.197614 | 1.713611 |
| 2000 | 6 | 0.983487 | 2.696035 | 0.892630 | 1.508403 |
| 2000 | 7 | 1.130085 | 2.577774 | 0.889332 | 1.475991 |
| 2000 | 8 | 1.121037 | 2.655483 | 0.780851 | 1.457726 |
| 2000 | 9 | 1.095642 | 2.368190 | 0.717600 | 1.599039 |
| 2000 | 10 | 1.071955 | 2.335051 | 0.706269 | 1.938029 |
| 2000 | 11 | 1.085157 | 2.107371 | 0.682538 | 1.750910 |
| 2000 | 12 | 1.027847 | 1.987937 | 0.795954 | 1.765470 |
| 2001 | 1 | 0.861586 | 2.318608 | 1.308786 | 1.525863 |
| 2001 | 2 | 0.774933 | 2.378520 | 1.220695 | 1.580994 |
| 2001 | 3 | 0.825737 | 2.341392 | 1.181483 | 1.890144 |
| 2001 | 4 | 0.801527 | 2.653348 | 1.031402 | 1.777031 |
| 2001 | 5 | 0.885553 | 2.626011 | 0.922292 | 1.584676 |
| 2001 | 6 | 0.812689 | 2.796778 | 0.822998 | 1.499585 |
| 2001 | 7 | 0.778799 | 2.644717 | 0.827703 | 1.494395 |
| 2001 | 8 | 0.908389 | 2.877797 | 0.759107 | 1.562994 |
| 2001 | 9 | 1.037934 | 2.485553 | 0.679648 | 1.991029 |
| 2001 | 10 | 0.939261 | 2.703040 | 0.673400 | 1.671858 |
| 2001 | 11 | 0.931589 | 2.456058 | 0.856708 | 1.497375 |
| 2001 | 12 | 0.905794 | 2.157420 | 0.877177 | 1.834960 |
| 2002 | 1 | 0.865642 | 2.088129 | 1.324053 | 1.936551 |
| 2002 | 2 | 0.902322 | 2.506011 | 1.269641 | 1.974696 |
| 2002 | 3 | 1.002809 | 2.597798 | 1.110135 | 1.706837 |
| 2002 | 4 | 0.874018 | 2.832530 | 1.010044 | 1.943313 |
| 2002 | 5 | 1.234767 | 2.723603 | 0.886578 | 1.862103 |
| 2002 | 6 | 1.078342 | 2.730930 | 0.812519 | 2.047204 |
| 2002 | 7 | 0.959816 | 2.794802 | 0.811802 | 1.806413 |
| 2002 | 8 | 1.008929 | 2.852671 | 0.687969 | 1.591838 |
| 2002 | 9 | 1.096069 | 2.634133 | 0.618372 | 1.439718 |
| 2002 | 10 | 1.182168 | 2.535121 | 0.690177 | 1.772926 |
| 2002 | 11 | 1.157880 | 2.406734 | 0.845681 | 1.835899 |
| 2002 | 12 | 1.006330 | 2.368127 | 1.062038 | 1.610423 |
| 2003 | 1 | 0.642172 | 2.613997 | 1.219597 | 1.910656 |
| 2003 | 2 | 0.628785 | 2.406454 | 1.127461 | 2.585198 |
| 2003 | 3 | 0.689655 | 2.512405 | 0.987696 | 2.275592 |
| 2003 | 4 | 0.580153 | 2.587534 | 0.914045 | 2.358429 |
| 2003 | 5 | 0.612655 | 2.278572 | 0.730425 | 2.294081 |
| 2003 | 6 | 0.657159 | 2.386553 | 0.614443 | 2.252357 |
| 2003 | 7 | 0.690227 | 2.196868 | 0.726967 | 2.163453 |
| 2003 | 8 | 0.781308 | 2.410332 | 0.724282 | 1.955484 |
| 2003 | 9 | 0.669060 | 2.418009 | 0.727042 | 1.879645 |
| 2003 | 10 | 0.687082 | 2.433634 | 0.739915 | 1.797848 |
| 2003 | 11 | 0.693647 | 2.334102 | 0.834537 | 1.811054 |
| 2003 | 12 | 0.707958 | 2.384079 | 0.857218 | 1.861597 |
| 2004 | 1 | 0.643716 | 1.955310 | 1.351843 | 1.908610 |
| 2004 | 2 | 0.626057 | 2.207397 | 1.193521 | 1.693527 |
| 2004 | 3 | 0.607310 | 2.470802 | 0.981958 | 2.040879 |
| 2004 | 4 | 0.587583 | 2.620431 | 0.740927 | 2.097122 |
| 2004 | 5 | 0.716138 | 2.797873 | 0.689491 | 1.682879 |
| 2004 | 6 | 0.792150 | 2.714561 | 0.882051 | 1.909566 |
| 2004 | 7 | 0.755586 | 2.649817 | 0.886335 | 1.480919 |
| 2004 | 8 | 0.719967 | 2.936795 | 0.875239 | 1.939849 |
| 2004 | 9 | 0.797805 | 2.740466 | 0.677619 | 1.843858 |
| 2004 | 10 | 0.750849 | 2.434177 | 0.840765 | 1.775612 |
| 2004 | 11 | 0.829484 | 2.450609 | 0.831019 | 1.939731 |
| 2004 | 12 | 0.781779 | 2.472085 | 0.939976 | 1.831405 |
| 2005 | 1 | 0.661328 | 2.233065 | 1.429365 | 1.841170 |
| 2005 | 2 | 0.640973 | 2.151476 | 1.049084 | 1.667907 |
| 2005 | 3 | 0.615243 | 2.124200 | 1.068880 | 1.617452 |
| 2005 | 4 | 0.580936 | 2.247145 | 0.930626 | 1.622666 |
| 2005 | 5 | 0.629283 | 2.154447 | 0.810973 | 1.381612 |
| 2005 | 6 | 0.663742 | 2.144862 | 0.832983 | 1.557797 |
| 2005 | 7 | 0.836655 | 2.206249 | 0.861328 | 1.738780 |
| 2005 | 8 | 0.812616 | 2.131328 | 0.862263 | 1.562843 |
| 2005 | 9 | 0.760273 | 2.263671 | 0.696948 | 1.325983 |
| 2005 | 10 | 0.789931 | 2.131625 | 0.749039 | 1.430152 |
| 2005 | 11 | 0.883357 | 2.073166 | 0.720527 | 1.396822 |
| 2005 | 12 | 1.000766 | 2.130619 | 0.828367 | 1.710556 |
| 2006 | 1 | 0.719862 | 1.955155 | 1.110376 | 1.874251 |
| 2006 | 2 | 0.649492 | 2.001666 | 0.978601 | 1.579375 |
| 2006 | 3 | 0.690928 | 1.985129 | 1.136483 | 1.412289 |
| 2006 | 4 | 0.622094 | 2.099810 | 1.093018 | 1.384627 |
| 2006 | 5 | 0.730895 | 2.288826 | 0.795519 | 1.381470 |
| 2006 | 6 | 0.700293 | 2.220143 | 0.764966 | 1.351906 |
| 2006 | 7 | 0.769778 | 2.081936 | 0.787064 | 1.184579 |
| 2006 | 8 | 0.797273 | 2.285233 | 0.726630 | 1.547498 |
| 2006 | 9 | 0.817381 | 2.324335 | 0.792386 | 1.609720 |
| 2006 | 10 | 0.871510 | 2.349371 | 0.964761 | 1.690291 |
| 2006 | 11 | 0.836253 | 2.325491 | 0.918828 | 1.458792 |
| 2006 | 12 | 0.844771 | 2.313429 | 0.886596 | 1.485768 |
| 2007 | 1 | 0.747894 | 2.059840 | 1.494106 | 1.302609 |
| 2007 | 2 | 0.652542 | 2.069661 | 1.429824 | 1.588480 |
| 2007 | 3 | 0.673634 | 2.021737 | 1.131848 | 1.491747 |
| 2007 | 4 | 0.635635 | 2.035813 | 1.154128 | 1.844690 |
| 2007 | 5 | 0.633732 | 2.054303 | 1.064528 | 1.769986 |
| 2007 | 6 | 0.752994 | 1.999358 | 0.884254 | 1.569659 |
| 2007 | 7 | 0.762981 | 1.842097 | 0.815854 | 1.767106 |
| 2007 | 8 | 0.825860 | 1.887843 | 0.659005 | 1.491909 |
| 2007 | 9 | 0.745552 | 1.862137 | 0.732431 | 1.400166 |
| 2007 | 10 | 1.002476 | 1.790210 | 0.832923 | 1.546036 |
| 2007 | 11 | 0.812479 | 1.922589 | 0.873921 | 1.918230 |
| 2007 | 12 | 0.920842 | 1.752011 | 0.986699 | 1.567522 |
| 2008 | 1 | 0.731479 | 1.821799 | 1.337323 | 1.398407 |
| 2008 | 2 | 0.647205 | 1.854689 | 1.321081 | 1.465372 |
| 2008 | 3 | 0.604773 | 1.853944 | 1.264385 | 1.324736 |
| 2008 | 4 | 0.659617 | 1.728667 | 1.019256 | 1.587139 |
| 2008 | 5 | 0.687588 | 1.914166 | 1.059509 | 1.644744 |
| 2008 | 6 | 0.756933 | 1.876961 | 0.972771 | 1.957292 |
| 2008 | 7 | 0.885538 | 1.741587 | 0.864564 | 1.800974 |
| 2008 | 8 | 0.901281 | 1.698459 | 0.736917 | 1.669752 |
| 2008 | 9 | 0.846034 | 1.745835 | 0.728946 | 1.726028 |
| 2008 | 10 | 1.039477 | 1.759273 | 0.925573 | 1.777882 |
| 2008 | 11 | 0.923398 | 1.721266 | 0.949022 | 2.222720 |
| 2008 | 12 | 0.923279 | 1.922899 | 0.966800 | 1.993928 |
| 2009 | 1 | 0.687855 | 1.796543 | 1.137879 | 1.529296 |
| 2009 | 2 | 0.620739 | 1.846300 | 1.064440 | 1.493151 |
| 2009 | 3 | 0.579204 | 1.781868 | 1.071533 | 1.411718 |
| 2009 | 4 | 0.632339 | 1.867064 | 0.863571 | 1.288615 |
| 2009 | 5 | 0.612332 | 1.818568 | 0.827208 | 1.634133 |
| 2009 | 6 | 0.799422 | 1.828982 | 0.857993 | 1.549561 |
| 2009 | 7 | 0.711187 | 1.639259 | 0.715279 | 1.273204 |
| 2009 | 8 | 0.782756 | 1.662778 | 0.777272 | 1.216928 |
| 2009 | 9 | 0.710586 | 1.632562 | 0.663890 | 1.130296 |
| 2009 | 10 | 0.853897 | 1.651060 | 0.862910 | 1.298955 |
| 2009 | 11 | 0.864294 | 1.782816 | 0.815109 | 1.178568 |
| 2009 | 12 | 0.818987 | 1.868954 | 0.780303 | 1.207391 |
| 2010 | 1 | 0.670465 | 1.762663 | 1.050857 | 0.952155 |
| 2010 | 2 | 0.654480 | 1.696722 | 1.061952 | 0.953518 |
| 2010 | 3 | 0.613193 | 1.756563 | 0.940568 | 1.138086 |
| 2010 | 4 | 0.600252 | 1.807910 | 0.782505 | 0.862918 |
| 2010 | 5 | 0.608718 | 1.559381 | 0.663539 | 0.773738 |
| 2010 | 6 | 0.621847 | 1.568924 | 0.703465 | 0.721336 |
| 2010 | 7 | 0.733771 | 1.598486 | 0.790545 | 1.018492 |
| 2010 | 8 | 0.734103 | 1.529946 | 0.735975 | 1.055412 |
| 2010 | 9 | 0.767330 | 1.567568 | 0.793326 | 1.018661 |
| 2010 | 10 | 0.741308 | 1.559445 | 0.858324 | 1.042197 |
| 2010 | 11 | 0.804106 | 1.705356 | 0.930226 | 1.093052 |
| 2010 | 12 | 0.794069 | 1.652478 | 0.988776 | 0.929378 |
| 2011 | 1 | 0.558350 | 1.552904 | 1.275934 | 0.664956 |
| 2011 | 2 | 0.507911 | 1.716854 | 1.048386 | 0.508621 |
| 2011 | 3 | 0.515845 | 1.662813 | 0.843555 | 0.445566 |
| 2011 | 4 | 0.519564 | 1.707393 | 0.795152 | 0.521630 |
| 2011 | 5 | 0.535953 | 1.574453 | 0.711085 | 0.557548 |
| 2011 | 6 | 0.645390 | 1.592223 | 0.753298 | 0.846919 |
| 2011 | 7 | 0.688549 | 1.651038 | 0.697699 | 0.942191 |
| 2011 | 8 | 0.729938 | 1.506264 | 0.652668 | 1.050501 |
| 2011 | 9 | 0.686085 | 1.577457 | 0.821426 | 1.006228 |
| 2011 | 10 | 0.649393 | 1.512957 | 0.887554 | 1.305759 |
| 2011 | 11 | 0.692826 | 1.684182 | 0.785292 | 1.213415 |
| 2011 | 12 | 0.641083 | 1.674603 | 0.959176 | 1.443763 |
| 2012 | 1 | 0.531460 | 1.710701 | 1.060092 | 1.260298 |
| 2012 | 2 | 0.499401 | 1.563314 | 1.025586 | 1.291859 |
| 2012 | 3 | 0.550425 | 1.562052 | 0.765383 | 1.192131 |
| 2012 | 4 | 0.522500 | 1.529692 | 0.648530 | 1.708855 |
| 2012 | 5 | 0.527375 | 1.682672 | 0.658745 | 1.522842 |
| 2012 | 6 | 0.571288 | 1.651958 | 0.721992 | 1.786046 |
| 2012 | 7 | 0.589974 | 1.649480 | 0.676412 | 1.563217 |
| 2012 | 8 | 0.632497 | 1.605226 | 0.603779 | 1.488866 |
| 2012 | 9 | 0.619684 | 1.484276 | 0.662611 | 1.816427 |
| 2012 | 10 | 0.620426 | 1.484270 | 0.756081 | 1.963241 |
| 2012 | 11 | 0.709196 | 1.490928 | 0.752606 | 2.016546 |
| 2012 | 12 | 0.718547 | 1.376533 | 0.690024 | 2.054678 |
| 2013 | 1 | 0.649495 | 1.530279 | 1.054653 | 1.846397 |
| 2013 | 2 | 0.517137 | 1.429419 | 0.840349 | 1.340601 |
| 2013 | 3 | 0.509963 | 1.535440 | 0.699162 | 1.276271 |
| 2013 | 4 | 0.486519 | 1.539660 | 0.659931 | 1.151168 |
| 2013 | 5 | 0.515740 | 1.506506 | 0.638782 | 1.291069 |
| 2013 | 6 | 0.551458 | 1.504155 | 0.658372 | 1.134686 |
| 2013 | 7 | 0.542668 | 1.689613 | 0.598921 | 1.009550 |
| 2013 | 8 | 0.571088 | 1.526227 | 0.507649 | 1.242699 |
| 2013 | 9 | 0.614444 | 1.543178 | 0.664192 | 1.253074 |
| 2013 | 10 | 0.618153 | 1.704420 | 0.790059 | 1.557294 |
| 2013 | 11 | 0.706081 | 1.813546 | 0.718618 | 1.697949 |
| 2013 | 12 | 0.695244 | 1.654914 | 0.899551 | 1.626338 |
| 2014 | 1 | 0.610342 | 1.758975 | 0.961199 | 1.054249 |
| 2014 | 2 | 0.554708 | 1.908621 | 0.939442 | 0.885212 |
| 2014 | 3 | 0.532215 | 1.807348 | 0.767672 | 0.925498 |
| 2014 | 4 | 0.515424 | 1.834965 | 0.632283 | 1.007303 |
| 2014 | 5 | 0.510298 | 1.813321 | 0.421464 | 1.036471 |
| 2014 | 6 | 0.587451 | 1.793765 | 0.568475 | 1.197558 |
| 2014 | 7 | 0.627759 | 1.712471 | 0.568996 | 0.938258 |
| 2014 | 8 | 0.665984 | 1.909652 | 0.502395 | 0.782027 |
| 2014 | 9 | 0.743663 | 1.835826 | 0.621328 | 1.007960 |
| 2014 | 10 | 0.632809 | 1.909194 | 0.815594 | 1.376197 |
| 2014 | 11 | 0.702087 | 1.877106 | 0.752774 | 1.468706 |
| 2014 | 12 | 0.737582 | 1.949118 | 0.777925 | 1.553833 |
| 2015 | 1 | 0.582459 | 1.954609 | 0.910905 | 1.068640 |
| 2015 | 2 | 0.569588 | 1.830295 | 0.954004 | 0.959471 |
| 2015 | 3 | 0.565218 | 1.686834 | 0.804438 | 0.927746 |
| 2015 | 4 | 0.520593 | 1.694663 | 0.640808 | 0.994226 |
| 2015 | 5 | 0.526804 | 1.711197 | 0.564567 | 0.992867 |
| 2015 | 6 | 0.603091 | 1.786478 | 0.466618 | 0.999871 |
| 2015 | 7 | 0.753735 | 1.741796 | 0.630781 | 1.015220 |
| 2015 | 8 | 0.692710 | 1.785508 | 0.560337 | 1.151509 |
| 2015 | 9 | 0.651039 | 1.588454 | 0.676165 | 1.163341 |
| 2015 | 10 | 0.658400 | 1.551410 | 0.689920 | 1.309952 |
| 2015 | 11 | 0.676442 | 1.615653 | 0.532037 | 1.302648 |
| 2015 | 12 | 0.792865 | 1.742606 | 0.615219 | 1.266029 |
| 2016 | 1 | 0.680092 | 1.566900 | 0.980987 | 1.155393 |
| 2016 | 2 | 0.621800 | 1.659699 | 0.856309 | 0.823886 |
| 2016 | 3 | 0.538960 | 1.673425 | 0.609534 | 0.918021 |
| 2016 | 4 | 0.607400 | 1.367955 | 0.488195 | 0.792955 |
| 2016 | 5 | 0.603741 | 1.444645 | 0.508428 | 0.837839 |
| 2016 | 6 | 0.603170 | 1.524195 | 0.414037 | 0.771329 |
| 2016 | 7 | 0.682015 | 1.636899 | 0.535659 | 0.667788 |
| 2016 | 8 | 0.616559 | 1.476790 | 0.514522 | 0.758434 |
| 2016 | 9 | 0.624286 | 1.438380 | 0.635916 | 0.922363 |
| 2016 | 10 | 0.616275 | 1.531038 | 0.793296 | 1.403971 |
| 2016 | 11 | 0.601919 | 1.557787 | 0.831309 | 1.613462 |
| 2016 | 12 | 0.604437 | 1.665212 | 0.790473 | 1.055286 |
| 2017 | 1 | 0.510598 | 1.697453 | 0.892477 | 0.908781 |
| 2017 | 2 | 0.468869 | 1.469725 | 0.856417 | 0.846476 |
| 2017 | 3 | 0.541431 | 1.394861 | 0.650903 | 0.793476 |
| 2017 | 4 | 0.553842 | 1.394627 | 0.392111 | 0.643227 |
| 2017 | 5 | 0.607912 | 1.550311 | 0.435648 | 0.928095 |
| 2017 | 6 | 0.581797 | 1.554132 | 0.434582 | 0.797279 |
| 2017 | 7 | 0.558668 | 1.583194 | 0.451652 | 0.811581 |
| 2017 | 8 | 0.602439 | 1.592248 | 0.515115 | 0.902124 |
| 2017 | 9 | 0.566921 | 1.527640 | 0.613566 | 0.891694 |
| 2017 | 10 | 0.536949 | 1.442264 | 0.771232 | 0.977127 |
| 2017 | 11 | 0.558542 | 1.361620 | 0.874257 | 0.984499 |
| 2017 | 12 | 0.558575 | 1.493027 | 0.783506 | 1.117038 |
| 2018 | 1 | 0.448128 | 1.548706 | 0.857144 | 0.927990 |
| 2018 | 2 | 0.423191 | 1.517504 | 0.875634 | 0.830860 |
| 2018 | 3 | 0.442817 | 1.484574 | 0.687378 | 0.752620 |
| 2018 | 4 | 0.447212 | 1.599099 | 0.639848 | 0.784222 |
| 2018 | 5 | 0.455717 | 1.488744 | 0.499104 | 0.853188 |
| 2018 | 6 | 0.473864 | 1.509313 | 0.496301 | 0.845470 |
| 2018 | 7 | 0.539689 | 1.462994 | 0.590652 | 0.945627 |
| 2018 | 8 | 0.490840 | 1.536642 | 0.613756 | 1.030970 |
| 2018 | 9 | 0.496483 | 1.466003 | 0.665353 | 0.963637 |
| 2018 | 10 | 0.488842 | 1.623338 | 0.816713 | 0.865506 |
| 2018 | 11 | 0.479451 | 1.568070 | 0.742712 | 0.939014 |
| 2018 | 12 | 0.488588 | 1.519539 | 0.725393 | 0.925111 |
